# Novel non‐invasive molecular signatures for oral cavity cancer, by whole transcriptome and small non‐coding RNA sequencing analyses: Predicted association with PI3K/AKT/mTOR pathway

**DOI:** 10.1002/cam4.7309

**Published:** 2024-05-31

**Authors:** Dimitra P. Vageli, Panagiotis G. Doukas, Jeffrey P. Townsend, Curtis Pickering, Benjamin L. Judson

**Affiliations:** ^1^ Yale Larynx Lab, Surgery Otolaryngology Yale School of Medicine New Haven Connecticut USA; ^2^ Department of Medicine Saint Peter's University Hospital/Rutgers‐ RWJ Medical School New Brunswick New Jersey USA; ^3^ Department of Biostatistics Yale School of Public Health New Haven Connecticut USA; ^4^ Department of Surgery, Division of Otolaryngology Yale Medical School New Haven Connecticut USA

**Keywords:** biomarkers, miRNA, mRNA, oral cancer, oral squamous cell carcinoma, PI3K/AKT/mTOR, small non‐coding RNA sequencing, whole transcriptome sequencing

## Abstract

**Introduction:**

Identification of molecular biomarkers in the saliva and serum of oral cavity cancer patients represents a first step in the development of essential and efficient clinical tools for early detection and post‐treatment monitoring. We hypothesized that molecular analyses of paired saliva and serum samples from an individual would likely yield better results than analyses of either serum or saliva alone.

**Materials and Methods:**

We performed whole‐transcriptome and small non‐coding RNA sequencing analyses on 32 samples of saliva and serum collected from the same patients with oral squamous cell carcinoma (OSCC) and healthy controls (HC).

**Results:**

We identified 12 novel saliva and serum miRNAs and a panel of unique miRNA and mRNA signatures, significantly differentially expressed in OSCC patients relative to HC (log2 fold change: 2.6**–**26.8; DE: 0.02**–**0.000001). We utilized a combined panel of the 10 top‐deregulated miRNAs and mRNAs and evaluated their putative diagnostic potential (>87% sensitivity; 100% specificity), recommending seven of them for further validation. We also identified unique saliva and serum miRNAs associated with OSCC and smoking history (OSCC smokers vs. never‐smokers or HC: log2 fold change: 22–23; DE: 0.00003–0.000000001). Functional and pathway analyses indicated interactions between the discovered OSCC‐related non‐invasive miRNAs and mRNAs and their targets, through PI3K/AKT/mTOR signaling.

**Conclusion:**

Our data support our hypothesis that using paired saliva and serum from the same individuals and deep sequencing analyses can provide unique combined mRNA and miRNA signatures associated with canonical pathways that may have a diagnostic advantage relative to saliva or serum alone and may be useful for clinical testing. We believe this data will contribute to effective preventive care by post‐treatment monitoring of patients, as well as suggesting potential targets for therapeutic approaches.

## 1 INTRODUCTION

Oral squamous cell carcinoma (OSCC) is associated with poor survival if not diagnosed early. Based on recent preclinical findings, cancer‐related mRNA and miRNA expression changes occur early during head and neck carcinogenesis.^1^, ^2^, ^3^, ^4^, ^5^ These expression changes can involve the deregulation of cancer‐related molecules, such as miRNA markers, as well as inflammatory or cancer‐related factors and their related oncogenic signaling pathways.^5^, ^6^ Histopathologic examination of a tissue biopsy is the gold‐standard diagnostic approach for detecting oral malignant and premalignant lesions. However, its utility as a biomarker for early detection and risk assessment of malignant lesions is limited by several factors, such as morbidity of oral biopsy, time and resource requirements, the risk of sampling bias, and the potential for underdiagnoses.^7^ Therefore, rapid approaches that are sensitive, specific, and less invasive would improve the early diagnosis and prognosis of oral malignancy, especially in patients with known risk factors like a history of smoking. Such approaches would also aid in monitoring the effectiveness of treatment and in disease surveillance following successful treatment of patients with malignant lesions.^8^, ^9^, ^10^

Since 2020, there has been an increasing interest in the exploration of non‐invasive miRNA biomarkers in head and neck cancer (HNC).^8^, ^10^, ^11^ Park and colleagues^12^ were the first to perform an extended study of miRNAs in the saliva of HNC patients and healthy controls (HCs), followed by Salazar,^13^ Momen‐Heravi,^14^ Duz,^15^ Romani,^16^ and their colleagues. However, there is limited data available from sequencing analyses of whole saliva and paired serum from the same patient with oral cancer and HCs.^10^ In addition, there is a limited prior study using whole‐transcriptome analysis in saliva or serum to discover mRNA and miRNA profiles in OSCC patients. Our recent pilot study, analyzing 20 HNC‐related miRNAs in the saliva of oral cavity cancer patients and healthy individuals, by qPCR analysis, demonstrated an association between four miRNAs and oral cancer.^17^ By that smaller‐scale success, a more powerful whole‐transcriptome and/or small RNA sequencing analyses would potentially discover novel non‐invasive molecular biomarkers associated with OSCC,^14^, ^18^, ^19^, ^20^ enable assessment of the risk of patients for progression or recurrence, and identify biomarkers for future study as potential clinical tools.

We hypothesized that deep sequencing analysis would reveal unique and novel saliva and serum mRNA and miRNA profiles associated with OSCC and canonical oncogenic pathways. We also hypothesized that these signatures may differ between never‐smoking patients and patients with a smoking history. To investigate this hypothesis, we analyzed for the first time by whole‐transcriptome (mRNA‐seq) and small non‐coding RNA (smRNA‐seq) sequencing analyses paired saliva and serum from the same oral cavity patients and healthy individuals of smokers and never‐smokers and performed functional and pathway analyses. Our results provide promise for the development of a successful combined panel of non‐invasive molecular biomarkers associated with oral cavity cancer for post‐monitoring of patients and target therapy approaches.

## 2 METHODS

### 2.1 Patients

We collected 32 saliva and serum samples from 16 participants, including eight oral cancer patients and eight healthy individuals (Yale New Haven Hospital), using previously established collection methods^8^, ^9^, ^17^ and described in supplementary methods. All participants provided oral and written consent for participation in this study and donation of saliva and serum samples to the Yale Head and Neck Biorepository according to the Yale IRB‐approved protocol (HIC#1206010419). Histopathologic information and other characteristics of each participant for both study and control groups are shown in Table S1.

### 2.2 Sequencing and data analyses

We isolated total RNA from saliva and serum and performed whole‐transcriptome analysis and small non‐coding RNA sequencing, including miRNAs, according to previously established protocols (Supplementary methods).^17^ Samples were analyzed by YCGA (Yale Center for Genome Analysis) using Illumina NovaSeqX™ plus (Illumina, USA).^21^, ^22^ We identified mRNA and miRNA transcripts that were upregulated or downregulated significantly (adjusted *p* < 0.05) by DESeq2 software^23^, ^24^ in saliva and serum study groups of OSCC compared to HCs and assessed the gene‐expression patterns associated with OSCC and smoking history.

We evaluated the individual and combined sensitivity and sensitivity of up to 10 top saliva and serum miRNAs and mRNAs (>70%) by examination of the receiver operating characteristic curve (ROC curve, by GraphPad Prism 9.0) and as previously described and in Table S2.^25^, ^26^

### 2.3 Biological role

We used miRbase, miRDB, miRTarBase, and TarBase1,^27^, ^28^ to predict the biological role of identified miRNAs using their target identification. We also used functional annotation analysis and pathway analysis (Ingenuity Pathway Analysis; IPA, by Qiagen) to predict the biological role of differentially expressed genes, as well as interactions between miRNAs and mRNA saliva and serum differentially expressed molecules. Specifically, we performed comparison analysis (ore analysis), Biomarker Filter Results (human, mouse, rat), Pathway analysis (canonical pathways, upstream, diseases and disorders, such as cancer/head and neck cancer/oral cancer, networks, downstream effect analysis, target prediction analysis, etc.) on expression levels (Log ratio, decreasing *z*‐score < −1.6 or increasing *z*‐score >2.0; *p*_adj_ value ≤0.05).

### 2.4 cDNA synthesis, qPCR and immunohistochemical analysis (IHC)

We performed reverse transcription (RT) of total RNA for cDNA synthesis and qPCR for *RELA* mRNAs (*hGAPDH*: reference control gene; Qiagen Inc.) in seven serum OSCC and seven serum HC of our cohort, as well as IHC analysis for NF‐κB in Formalin‐fixed, and paraffin‐embedded (FFPE) tissue sections from eight OSCC tumors of our cohort relative to adjacent non‐pathologic tissues (ANTs), such as regional negative for invasion lymph nodes, retrieved from the Pathology archive (Yale Pathology), [NF‐*κ*B p65 (F‐6); Santa Cruz Biotechnology., Inc., Europe], as we previously described and in supplementary methods.^1^, ^2^, ^3^, ^4^, ^5^, ^6^

## 3 RESULTS

### 3.1 Novel and unique saliva and serum miRNAs associated with OSCC and smoking history

We analyzed the highest number of miRNAs so far (2731 miRNAs) in the saliva and serum of OSCC patients and HC compared to prior studies^12^, ^13^, ^14^, ^15^, ^16^ and detected the highest number of expressed miRNAs (1370 unique miRNAs for OSCC). We present for the first time a panel of seven unique miRNAs, four in saliva and three in serum, associated with OSCC (Table S3),^29^, ^30^, ^31^ except miR‐23a, which was previously reported by a single study by qPCR at reduced levels in the serum of benign salivary gland neoplasms relative to controls.^32^ Based on our data, saliva and serum demonstrated distinct miRNA profiles associated with OSCC.

#### 3.1.1 Saliva miRNAs

**FIGURE 1:**
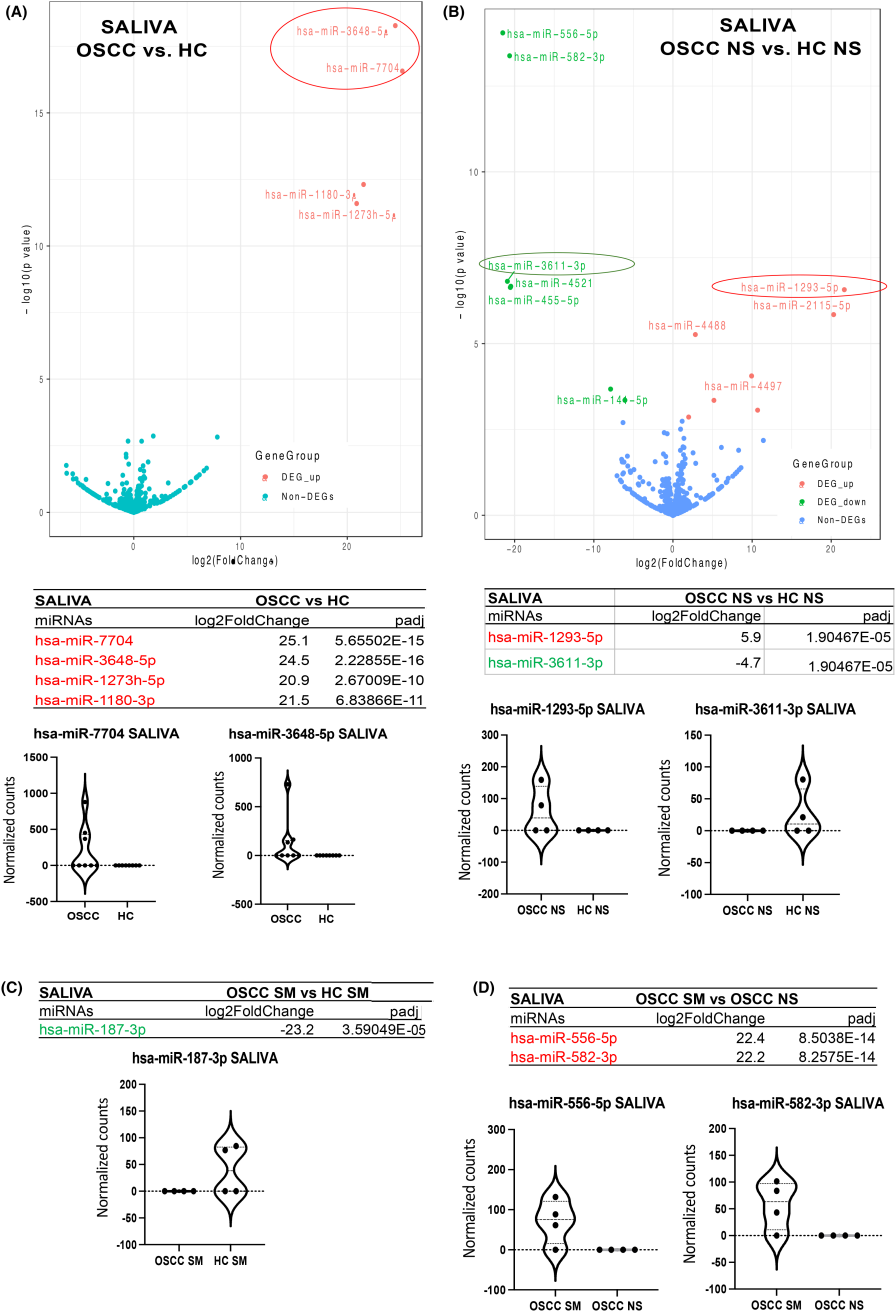
Saliva contained unique miRNAs differentially expressed in (A) OSCC patients versus HC, (B) OSCC NS (never smokers) versus HC NS, (C) OSCC SM (smokers) versus HC SM, (D) OSCC SMs versus OSCC NS red: upregulation; green: downregulation; volcano plot, log_2_ fold‐change and *p*_adj_ values were obtained by DESeq2; violin graphs depict normalized miRNA counts of each markers per group; by GraphPad Prism 9.0 software.

Our analysis by small non‐coding RNA sequencing (smRNA‐seq) revealed 22 unique miRNAs differentially expressed in the saliva of OSCC patients relative to HC (*p* < 0.05; Table S4A), four of those demonstrated the most significant differential expression changes (fold‐changes and *p* values) in OSCC versus HC (Figure 1A). Specifically, stem‐loop hsa‐miR‐7704, hsa‐miR‐3648‐5p, hsa‐1273 h‐5p, and hsa‐1180‐3p demonstrated the highest levels of significant overexpression (20.9–25.2 log_2_ fold‐change; *p*_adj_≤0.0001; by DESeq2) in OSSC compared to HC (Figure 1A).

Specifically, in never‐smokers, 14 miRNAs showed statistically significant deregulation in the saliva of OSCC versus HC (2.0–21.5 log_2_ fold‐change; *p*_adj_ *≤* 0.05 (Table S4B). Hsa‐miR‐1293‐5p and hsa‐miR‐3611‐3p, demonstrated the highest levels of significant overexpression and downregulation, respectively, in this group (20.9–21.7 log_2_ fold‐change; *p*_adj_ *≤* 0.0001) (Figure 1B). In smokers, hsa‐miR‐187‐3p was significantly downregulated in the saliva of OSCC versus HC (23.2 log_2_ fold‐change; *p*_adj_ *≤* 0.0001) (Figure 1C). When comparing smokers to never‐smokers, 13 miRNAs were significantly differentially expressed in the saliva of OSCC smokers compared to OSCC never‐smokers (2.2–22.4 log_2_ fold‐change; *p*_adj_ <<0.01) (Table S4B). Hsa‐582‐3p and hsa‐miR‐556‐5p demonstrated the highest levels of overexpression in the saliva of this group (22.2–22.4 log_2_ fold‐change; *p*_adj_ <<0.0001) (Figure 1D).

#### 3.1.2 Serum miRNAs

**FIGURE 2:**
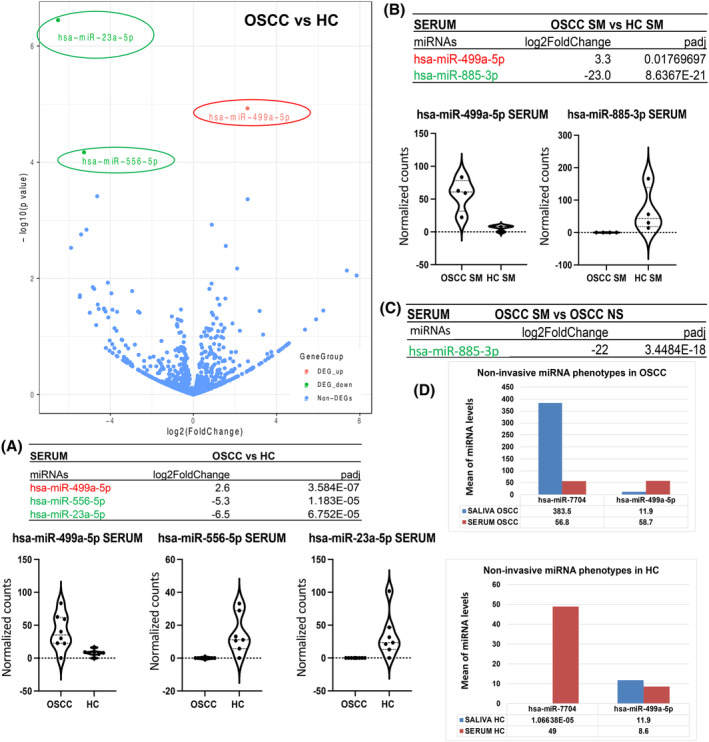
Serum contained unique miRNAs differentially expressed in (A) OSCC patients versus HC, (B) OSCC smokers versus HC smokers, (C) OSCC smokers versus OSCC never‐smokers, (D) Saliva and serum hsa‐miR‐7704 and hsa‐miR‐499‐5p phenotypes in OSCC patients and HC [red: upregulation; green: downregulation; volcano plot, log_2_ fold‐change and *P*_adj_ values were obtained by DESeq2; violin graphs depict normalized miRNA counts of each marker per group; by GraphPad Prism 9.0 software.

Our analysis by smRNA‐seq revealed 43 unique miRNAs differentially expressed in the serum of OSCC patients relative to HC (*p* < 0.05; Table S4A), four of those demonstrated the most significant differential expression changes (fold‐changes and *p* values) in OSCC versus HC (Figure 2A). Specifically, stem‐loop hsa‐miR‐499a‐5p, hsa‐miR‐23a‐5p, and hsa‐miR‐556‐5p were found to be statistically significantly altered (by DESeq2) in the serum of OSSC patients versus HC (2.3–6.5 log_2_ fold‐change; *p*_adj_ = <0.0001), particularly miR‐499a‐5p in smokers with OSCC (3.3 log_2_ fold‐change; *p*_adj_ <<0.02) (Figure 2B; Table S4B). Also, hsa‐miR‐885‐3p demonstrated statistically significant reduced levels in the serum of smokers with OSCC compared to HC smokers or compared to OSCC never‐smokers (21.7–23.0 log_2_ fold‐change; *p*_adj_ <<0.0001) (Figure 2B,C; Table S4B). Our analysis revealed an inverted molecular phenotype between saliva and serum molecules in OSCC and HC, as shown in Figure 2D, for the two most altered miRNAs. Specifically, our analysis revealed increased miR‐7704 and decreased miR‐449a‐5p in the saliva of OSCC patients compared to their serum. On the contrary healthy individuals demonstrated decreased miR‐7704 and increased miR‐499a‐5p in their saliva compared to their serum.

#### 3.1.3 Biological role‐Target prediction miRNA analysis

Our analysis predicted that both saliva and serum miRNAs associated with OSCC have significant biological roles (Table 1A **)**, particularly through the PI3K/AKT pathway, which is associated with HNC (Table 1B).^33^ Our analysis also predicted that identified saliva and serum miRNAs target molecules that are also targets of drugs (Table 1B). Specifically, target prediction miRNA analysis revealed that six of the differentially expressed saliva and serum miRNAs, including tobacco smoked‐related miR‐187‐3p, can target genes associated with an inflammatory and cancer‐related or antiapoptotic function through cancer‐related canonical pathways, such as PI3K/AKT/mTOR, MAP3K8, BCL2, DGKZ/mTOR (Table 1B).^33^, ^34^, ^35^, ^36^, ^37^

**TABLE 1:** (A) Biological role of unique saliva and serum miRNAs associated with OSCC and (B) their predicted interactions with saliva and serum mRNAs.

A. Biological role of miRNAs associated with OSCC			
miRNA (log_2_ fold change, *p*_adj_)	Location	Predicted target	Function
Saliva			
miR‐7704 (25.2, <0.0001)	Cytoplasm	TSC1/2 (negative regulator of mTOR1)	Cell growth
		SFN (stratifin)	Antiapoptosis
miR‐3648‐5p 24.5, <0.0001)	Cytoplasm	CCNF cyclin F	Cell growth
miR‐1180‐3p (21.5, <0.0001)	Cytoplasm	BAD (BCL2 associated agonist cell death)	Cell survival
miR‐1273 h‐5p (20.9, <0.0001)	Cytoplasm	TSC1/2 (complex tubericus sclerosis)	Cell growth
		CDKN1A (inhibitor of cell cycle)	Cell prolifedration
		MAPK8P1 (prevents MAPK8 activation)	Cell growth
Serum			
miR‐499‐5p (2.6, 0.0048)	Cytoplasm	CDKN1A (inhibitor of cell cycle)	Cell prolifedration
miR‐23a‐5p (−6.5, 0.00029)	Cytoplasm	THEM4 (Negative regulator of PI3K)	Cell proliferation
miR‐556‐5p (−5.3, 0.02)	Cytoplasm	IL18R1, IL6ST, BCL2L2‐PABPN1	Cell survival

B. Predicted interactions of miRNA with mRNA associated with OSCC					
Saliva—OSCC versus HC—PI3K/AKT signaling					
miRNA (log2FC, *p*_adj_ value)	Location	Predicted target	Function	Pathway	^a^Drugs
miR‐7704 (25.1, <0.0001)	Cytoplasm	TSC1/2 (negative regulator of mTOR1)/inhibition	Cell growth	PI3K/mTOR	Bimiralisib
		SFN (stratifin)/inhibition	Antiapoptosis	AKT/mTOR	Capivasertib
miR‐1273 h‐5p (20.9, <0.0001)	Cytoplasm	TSC1/2 (complex tuberous sclerosis)/inhibition	Cell growth	AKT/mTOR	Cativasertib
		CDKN1A (inhibitor of cell cycle) / inhibition	Cell proliferation	PI3K/AKT/mTOR	Idelalisib/rituximab
		MAPK8P1 (prevents MAPK8 activation) / Inhibition	Cell growth	MAP3K8	COT inhibitor‐2
miR‐1180‐3p (21.5, <0.0001)	Cytoplasm	BAD (BCL2 associated agonist cell death) / Inhibition	Cell survival	BCL2	Paclitaxel/ Pembrolizumab
Saliva—OSCC SS versu HC SS—mTOR signaling					
miR‐187‐3p (−23.2, <0.0001)	Cytoplasm	DGKZ (Promotes mTORC1) / Activation	Cell Growth	DGKZ/mTOR	ASP1570
SERUM—OSCC vs. HC—PI3K/AKT signaling					
miR‐499‐5p (2.6, 0.0048)	Cytoplasm	CDKN1A (inhibitor of cell cycle) / inhibition	Cell proliferation	PI3K/AKT/mTOR	Idelalisib/rituximab
miR‐23a‐5p (−6.5, 0.00029)	Cytoplasm	THEM4 (Negative regulator of PI3K) Activation	Cell proliferation	AKT/mTOR	Capivasertib

*Note*: log_2_FCFC: log_2_foldchange; ^a^Drugs also affect miRNA targets, by IPA.

#### 3.1.4 Novel miRNAs

smRNA‐seq revealed 37 novel miRNAs that have never been described: 17 in saliva, and 20 in serum (Table 2; Table S5). In the serum of both OSCC and healthy subjects, six novel miRNAs were identified in saliva and five novel miRNAs were identified in serum.

**TABLE 2:** Novel miRNAs in saliva or serum of both OSCC and HC.

ID	Probability	Chr	Start pos.	End Pos.	Mature miRNA sequence	Read Count
Saliva						
1	0.999999	chr22	50191179	50191198	CGGCUGUCCAAGAAGAGGGC	35
2	0.990177	chr22	50191179	50191196	CGGCUGUCCAAGAAGAGG	13
3	0.989648	chr22	50191178	50191197	CCGGCUGUCCAAGAAGAGGG	11
4	0.968595	chr22	50191178	50191196	CCGGCUGUCCAAGAAGAGG	18
5	0.999999	chr3	52393993	52394013	CCUUCUCGAGCCUUGAGUGUG	46
6	0.985129	chr3	52394001	52394012	AGCCUUGAGUGU	39
Serum						
1	0.999999	chr1	26554542	26554561	GCAGCAAGGAAGGCAGGGGU	731
2	0.999999	chr8	1755133	1755153	UUGCUGGGAAAGGGAGAAGUU	14
3	0.999997	chr2	111135385	111135405	UUGUGUCCAGUUGUUGGGGGA	59
4	0.999984	chr8	1755134	1755152	UGCUGGGAAAGGGAGAAGU	15
5	0.985564	chr6	119823108	119823128	UGAGCAAGUGAAGUAUGUGGU	54
6	0.967191	chr1	26554543	26554561	CAGCAAGGAAGGCAGGGGU	55

### 3.2 Saliva and serum mRNAs associated with OSCC

We discovered 912 significantly differentially expressed genes in paired saliva and serum of OSCC patients compared to healthy individuals, overlapping with head and neck carcinoma genes in the datasets (Table 6). We identified the top 5 genes—*TNC*, *MMP‐10*, *TP63* in saliva and *RELA* (*p65*), and *TCAIM* in serum—associated with OSCC.

#### 3.2.1 Saliva mRNAs

**FIGURE 3:**
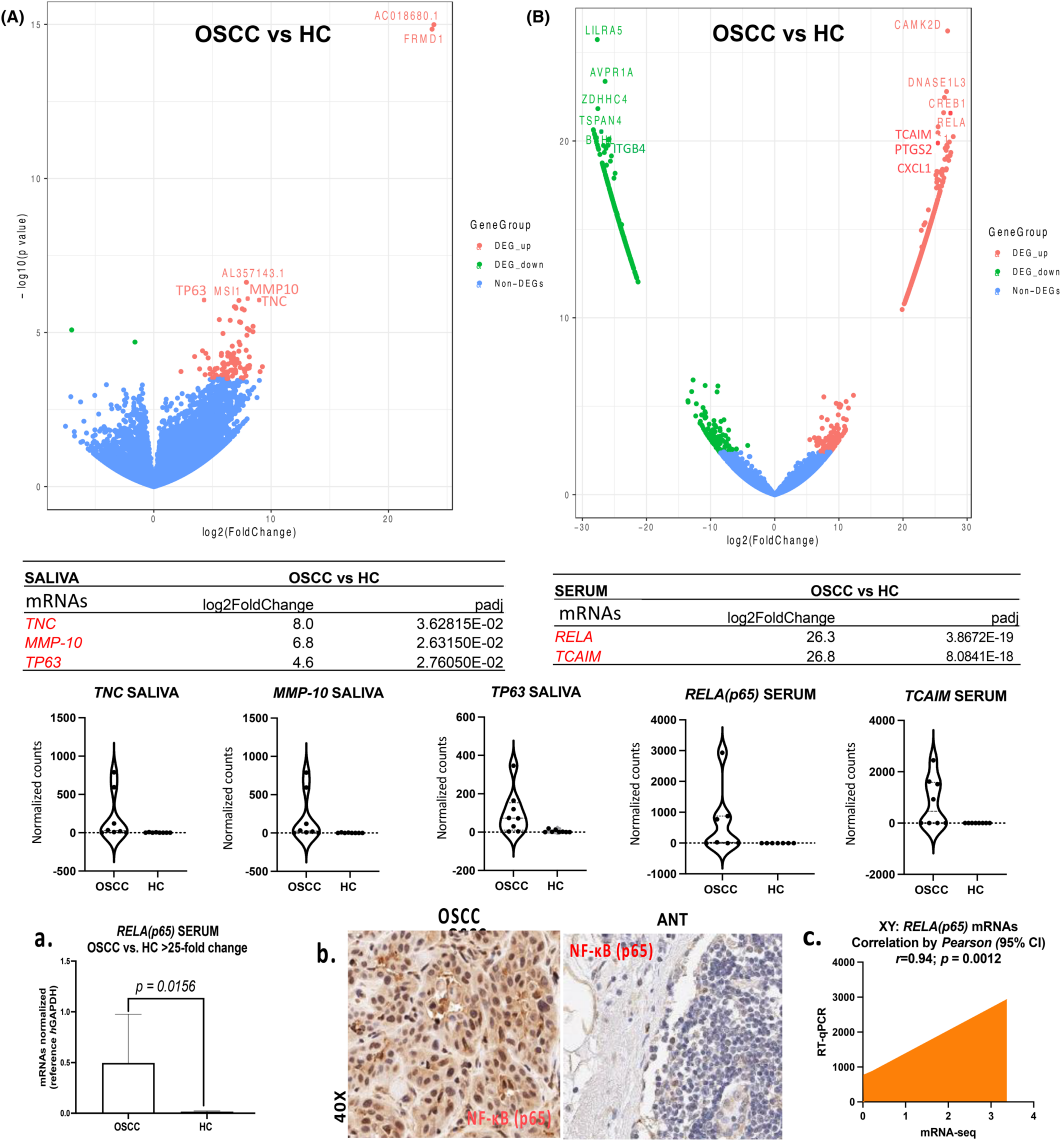
Saliva (A) and serum (B) contained unique mRNAs differentially expressed (DE) in OSCC patients versus HC. Volcano plots, log_2_ fold‐change, and *p*_adj_ values were obtained by DESeq2 (red: upregulation), while violin graphs depict normalized mRNA counts of each gene per group; by GraphPad Prism 9.0 software. (C) Elevated serum *RELA* (*p65*) mRNAs and NF‐κB tumor expression in OSCC. (a) The graph depicts increased levels of *RELA* (*p65*) mRNAs in the serum of OSCC patients versus HC of our cohort by qPCR (*p* < 0.05; by Wilcoxon Signed Rank Test). (b) Immunohistochemical analysis for NF‐κB in tumor specimens of our OSCC cohort showing strong nuclear staining relative to adjacent non‐pathologic tissue (ANT) (regional lymph node negative for invasion). (c) Graph by *Pearson* shows a significant linear correlation between *RELA* (*p65*) mRNA‐seq and RT‐qPCR analyses data of the serum from OSCC patients (*p* < 0.05; 95% CI: confidence interval; by GraphPad Prism 9.0).

Whole‐transcriptome (mRNA‐seq) and DESeq2 analysis revealed 102 significantly differentially expressed genes in the saliva of OSCC compared to HC (>2 log_2_ fold‐change; *p*_adj_ <0.05). We selected the top 32 deregulated genes (OSCC vs. HC: 5–24 log_2_ fold‐change; *p*_adj_ <0.0001) and identified their biological roles, such as tumor suppressor, cell proliferation, and cell–cell adhesion and migration function (Table S7A); pathway analysis (by IPA) revealed that three out of the top 32 deregulated genes—*TNC* (tenascin‐C), *MMP‐10*, and *TP63* (OSCC vs. HC: 5–8 log_2_ fold‐change; *p*_adj_ <0.05; Figure 3A), associated with canonical pathways, such as Tumor Microenvironment Pathway (Figure S1; Table S8A).

#### 3.2.2 Serum mRNAs

Whole‐transcriptome (mRNA‐seq) and DESeq2 analysis revealed 810 significantly differentially expressed genes in the serum of OSCC compared to HC (>2 log_2_ fold‐change; *p*_adj_ <0.05). We selected the top 32 deregulated genes (OSCC vs. HC: 23–28 log_2_ fold‐change; *p*_adj_ = <0.0001) and identified their biological roles, such as tumor suppressor, cell proliferation, and cell–cell adhesion and migration function (Table S7B), while pathway analysis (by IPA) revealed that nine out of the top 32 deregulated genes—*RELA*(*p65*), *PTGS2*, *CXCL1*, *ITGB4*, *CAMK2D*, *MPO*, *BAX*, *BRCA1* and *TCAIM* (OSCC vs. HC: 21–29 log_2_ fold‐change; *p*_adj_ <0.0001; Figure 3B)—were biologically significant and were associated with canonical pathways, such as IL‐8 signaling, PI3K/AKT Pathway, NF‐κB signaling, Molecular mechanism in Cancer, Tumor Microenvironment, or P53 signaling (Figure S2; Table S8B).

#### 3.2.3 Verification of NF‐κB overexpression in OSCC

RT‐qPCR analysis in the serum of OSCC patients and HC verified significant overexpression of *RELA* (*p65*) mRNAs in our cohort (OSCC vs. HC: 25‐fold change, *p* < 0.05; by *t*‐test) (Figure 3A,C; Table S9A). Also, immunohistochemical analysis for NF‐κB in OSCC tumor specimens documented a strong nuclear NF‐κB staining relative to their ANT (Figure 3B,C). *Pearson* analyses revealed a significant linear correlation between *RELA* (*p65*) mRNA‐seq and RT‐qPCR data of the serum from OSCC patients [*r* = 0.0946; *p* (two‐tailed) = 0.0012, by Pearson (95% confidence interval)] (Figure 3C; Table S9A).

These observations are consistent with our sequencing data showing that elevated *RELA* (p65) mRNAs in the serum can be a valuable biomarker for OSCC, which may reflect NF‐κB activation both in tumor cells and in the tumor microenvironment.

*Downstream effects analysis* (by IPA) revealed that the discovered saliva and serum mRNAs overlap with pathways and lists of head and neck carcinoma genes in the datasets and revealed the predicted effect of 50 saliva mRNAs on 268 downstream molecules (overlap *p* value 6.68 × 10^−3^; by IPA; Table S6A) and similarly, the predicted effect of 50 serum mRNAs on 246 downstream molecules (overlap *p* value 1.47 × 10^−6^; by IPA) (Table S6B).

*Target prediction mRNA analysis* revealed that saliva mRNA signatures are linked to oncogenic function targeting downstream molecules through the tumor microenvironment pathways (Figure S1). Based on IPA, *TNC* (tenascin‐C) and *MMP‐10* extracellular molecules can target molecules that are also targets of drugs, such as certolizumab, marimastat, iodine I 131 monoclonal antibodies, etc. (Table S8A). Our analysis also revealed that serum OSCC mRNA signatures can target genes associated with oncogenic canonical pathway signaling, such as those of IL‐8 (Figure S2), including several antiapoptotic and oncogenic factors, highlighting *PTGS2* and *RELA* upregulation, that can target other molecules that are also targets of drugs, such as etoricoxib and NF‐kB decoy (Table S8B).

### 3.3 Clustering of a combined panel of saliva and serum miRNA and mRNAs and hypothetical screening test for OSCC

**FIGURE 4:**
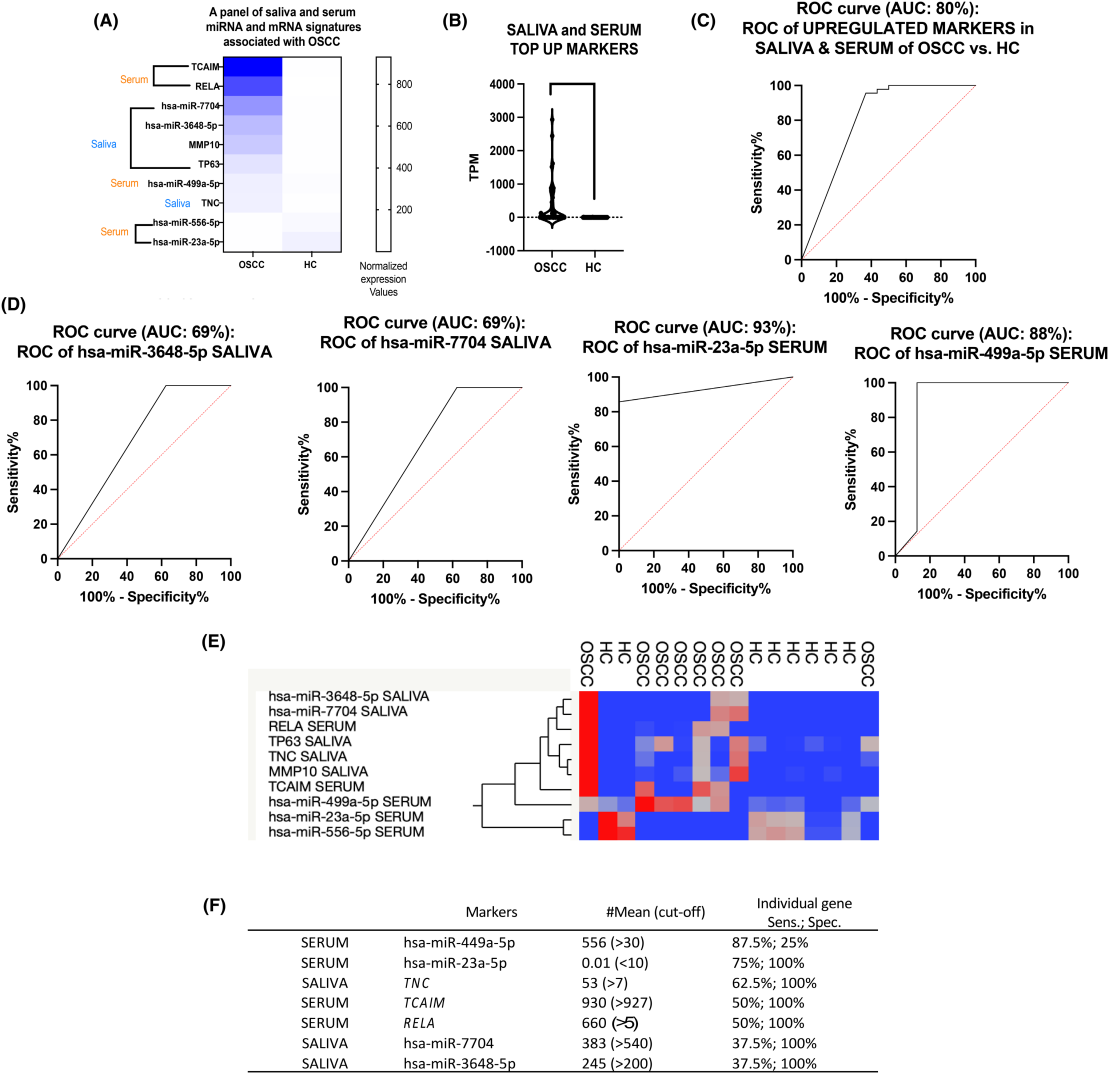
Evaluation of a panel of deregulated saliva and serum miRNAs and mRNAs in OSCC patients compared to healthy controls. (A) Heatmap created by GraphPadPrism 9.0 (paired *t*‐test *p*‐value = 0.0003). (B) The violin graph plot depicts the top upregulated combined saliva and serum markers (saliva miR‐7704, miR‐3648‐5p, *TNC*, and *MMP‐10*, serum *RELA* (*p65*) and T*CAIM*) (*p*‐value <0.0005, by *t*‐test). Graphs depict the ROC curve of all together (C) and each (D) of the top deregulated saliva and serum mRNAs and miRNAs; created by GraphPadPrism 9.0. (E) Heatmap created by JMP. (F) Seven proposed saliva and serum miRNA and mRNA markers for further evaluation. Sensitivity (Sens.), and specificity (Spec.) of each marker. #Mean, and cut‐off miRNA and mRNA levels (normalized counts by DESeq2).

We identified a panel of the top 10 saliva and serum miRNA and mRNA markers *TNC*, *MMP‐10*, *TP63*, miR‐7704, and miR‐3648 in saliva; and *RELA*, *TCAM1*, miR‐499a‐5p, miR‐23a‐5p, and miR‐556‐5p in serum, that were significantly differentially expressed in OSCC patients relative to healthy individuals (*t*‐test; *p* < 0.05; CI: 95%) (Figure 4A,B **)**, two of them, serum miR‐23a‐5p and miR‐449‐5p, with high sensitivity (>87%; by ROC curve) and five of them, saliva miR‐7704, miR‐3648, and *TNC*, and serum *TCAIM* and *RELA*, with high specificity (100%; Figure 4C,D Table S2). The heatmap created by JMP software shows the hierarchical clustering of expression of these markers in the different patients and healthy individuals (Figure 4E). Also, as shown in Figure 4F, miR‐449‐5p and miR‐23a‐5p were found to be sensitive markers in serum for screening OSCC relative to healthy individuals.

**FIGURE 5:**
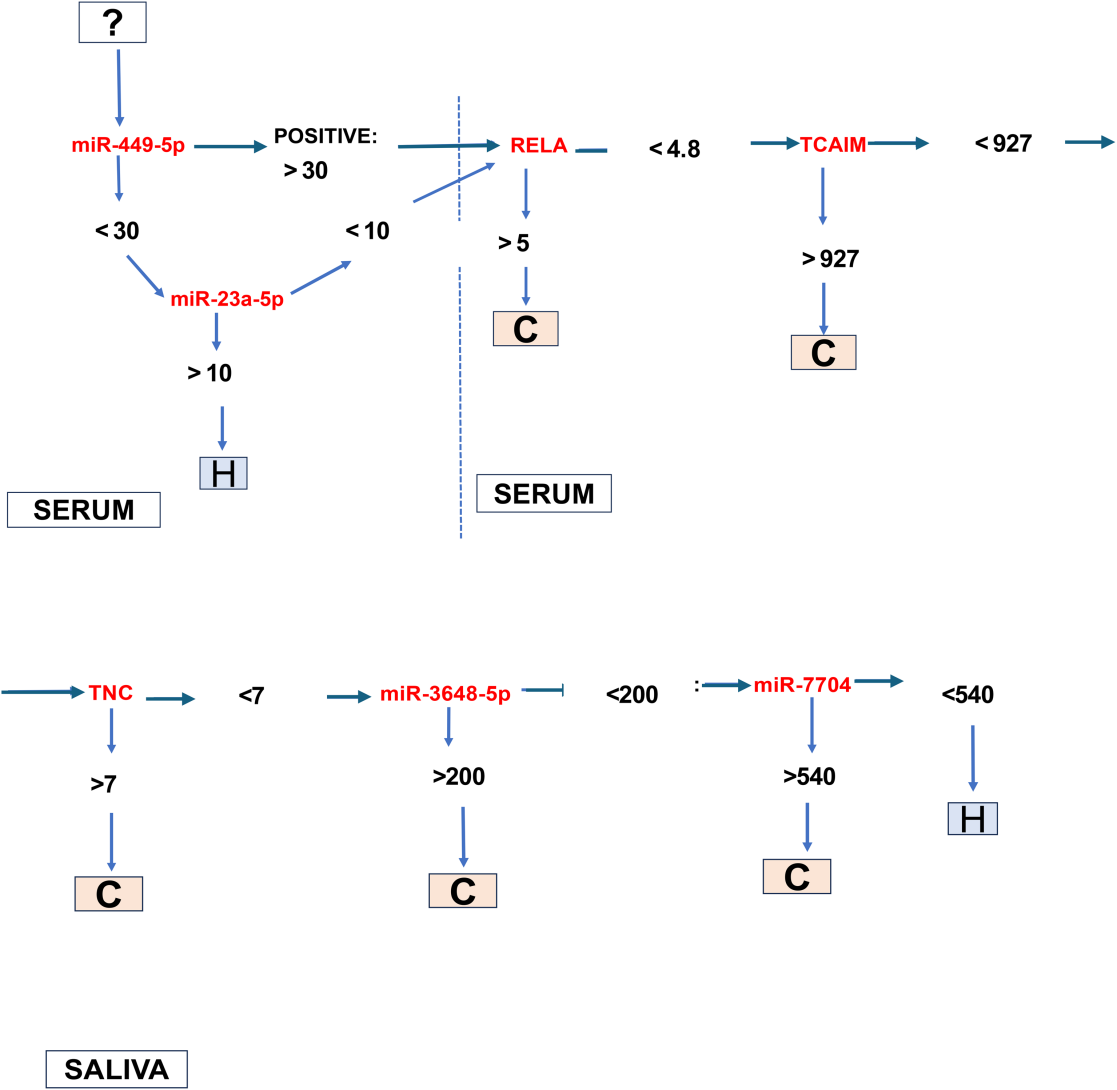
Hypothetical testing of saliva and serum miRNAs and mRNAs to screen patients with suspicious lesions for oral malignancy or after OSCC treatment at follow‐up, by sequencing analysis (values correspond to cut‐off normalized counts by DESeq2).

Based on the above, we selected serum miR‐449a‐55p which demonstrated the highest sensitivity (87.5%) and serum *RELA* (*p65*), *TCAIM*, and miR‐23a‐5p, as well as saliva *TNC*, miR‐3648‐5p, and miR‐7704 that demonstrated the highest specificity (100%) (Figure 4F **)**, over each saliva and serum miRNA or mRNA in a hypothetical screening test for OSCC (Figure 5). Specifically, as shown in Figure 5, a hypothetical screening test for OSCC would use serum miR‐499a‐5p as a sensitive marker to distinguish OSCC from healthy individuals, followed by serum miR‐23a‐5p *RELA* (*p65*), *TCAIM*, and saliva *TNC*, miR‐7704, and miR‐3648, biomarkers with high specificity. This set of serum and saliva molecules can be further clinically tested in an independent group of OSCC.

### 3.4 The biological role of saliva and serum miRNAs and mRNAs for OSCC

We performed pathway analysis to reveal the biological role of the discovered non‐invasive miRNAs in HNC by predicting miRNA targets and their interactions with the discovered mRNAs through cancer‐related canonical pathways. Identification of miRNA and mRNA networks through cancer‐related pathways and miRNA targets would support that the discovered non‐invasive molecular signatures represent future therapeutic targets or candidate biomarkers for monitoring the effectiveness of targeted therapy for oral cancer.

#### 3.4.1 Pathway analysis for saliva miRNAs and mRNAs

**FIGURE 6:**
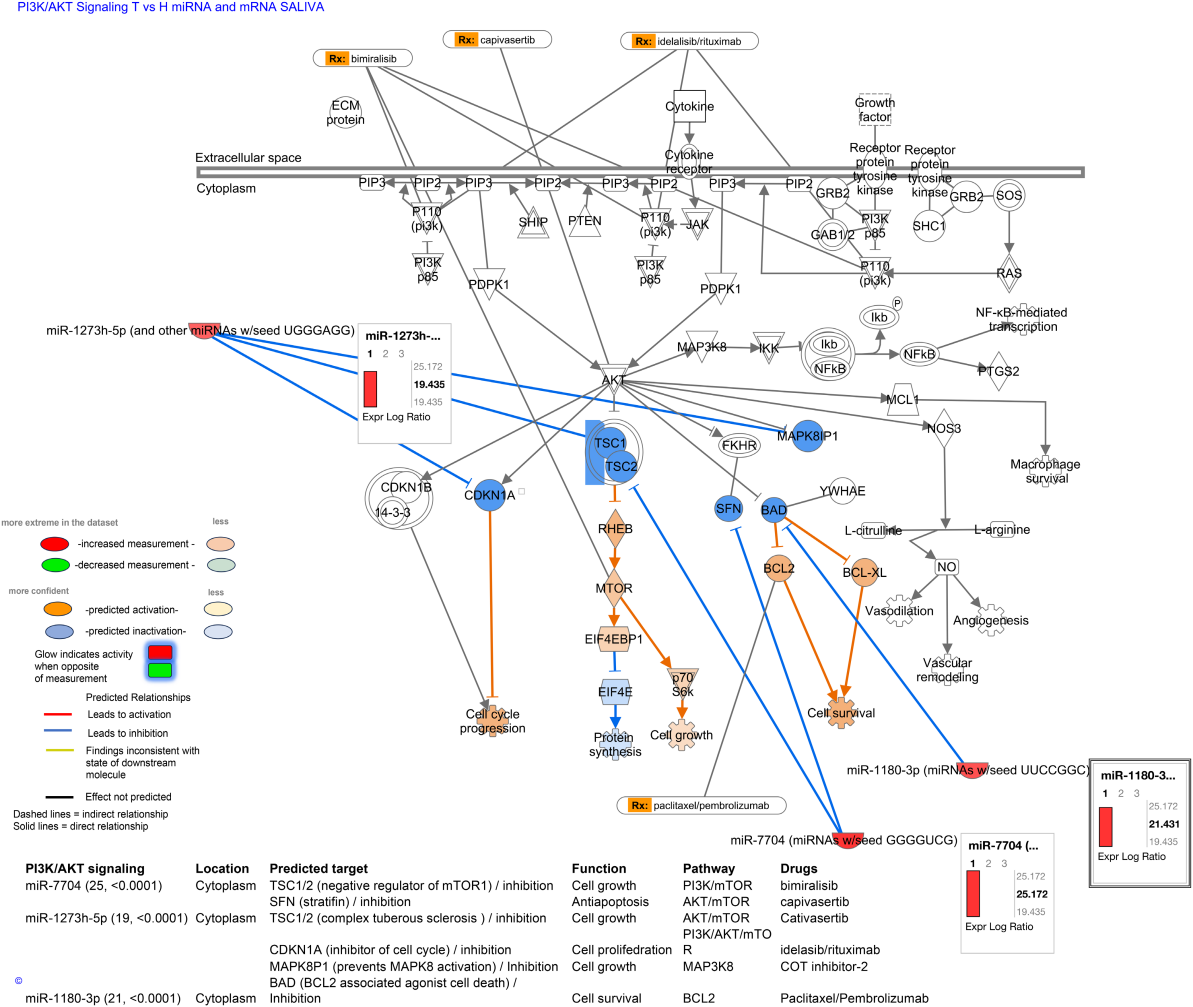
The diagram represents the predicted interactions between saliva miRNAs and saliva mRNAs, associated with OSCC, through the PI3K/AKT canonical pathway and their predicted targets, by IPA. The table presents saliva miRNAs, their location, predicted targets, function, pathway, and drugs that can also affect their targets.

Pathway analysis for saliva miRNAs and mRNAs revealed the predicted interactions of the unique saliva OSCC miRNAs, miR‐7704, miR‐1273 h‐5p, and miR‐1180‐3p can with the saliva‐identified mRNAs through PI3K/AKT signaling, and miRNA targets, including *TSC1*/*2*, *SFN*, *CDKN1A*, *MNAPK8P1*, and *BAD*, are also drug targets (Table 1B; Figure 6). Specifically, pathway analysis revealed that miR‐7704, can inhibit targets, including TSC1/2, which is a negative regulator of mTOR1 to promote cell growth,^33^, ^34^, ^35^ and SFN or stratifin factor, which is associated with cancer treatment resistance and PI3K/AKT signaling.^38^ Thus, based on our data miR‐7704 potentially control PI3K/AKT/mTOR pathway, similarly to drugs known such as bimiralisib and capivasertib that affect AKT and MTOR factors.^39^ Also, pathways analysis revealed that saliva miR‐1273 h‐5p and miR‐1180‐3p can interact with the discovered saliva mRNA profiles to regulate PI3K/AKT/mTOR pathway, the oncogene MAP3K8, which can activate downstream molecules including cancer‐related MEK, ERK, and JNK, NF‐κB, TNF, and IL‐1,^36^, ^40^ as well as BCL2 pathways.^37^ Based on our data, saliva miR‐1273 h‐5p and miR‐1180‐3p can target other molecules similarly, to known drugs, such as pembrolizumab.^41^ Finally, our analysis showed that the unique saliva miR‐187‐3p of tobacco smoke‐related OSCC can potentially interact with saliva‐identified mRNAs of this group through mTOR signaling, and that miR‐187‐3p predicted target, *DGKZ*, is also a drug target^42^ (Figure S3 and Table S9B).

#### 3.4.2 Pathway analysis for serum miRNAs and mRNAs

**FIGURE 7:**
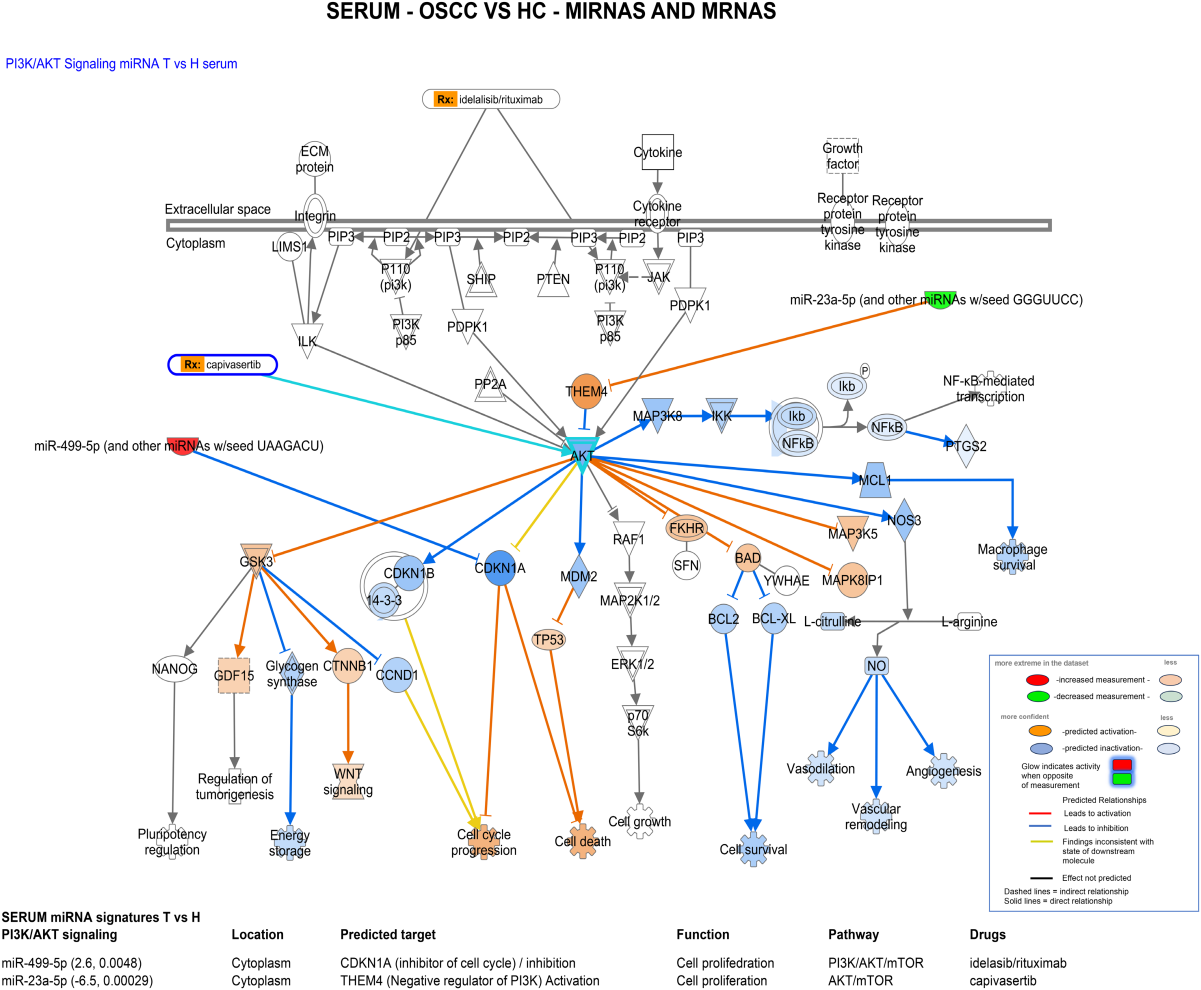
The diagram represents the predicted interactions between serum miRNAs and serum mRNAs (log_2_ fold‐change, and *p*_adj_ values), associated with OSCC, through the PI3K/AKT canonical pathway and their predicted targets, by IPA (by Qiagen).

Pathway analysis for serum miRNAs and mRNAs revealed that the unique serum OSCC miRNAs, miR‐499‐5p and miR‐23a‐5p, can potentially interact with serum‐identified mRNAs through PI3K/AKT signaling and their predicted targets, such as CDKN1A and THEM4, are also targets of drugs (Table 1B; Figure 7). Specifically, our analysis revealed that miR‐499‐5p can inhibit its targets, such as CDKN1A, which is a negative regulator of the cell cycle, to promote cell proliferation and therefore control PI3K/AKT/mTOR pathway, targeting other molecules that are targets of drugs such as idelalisib and rituximab. Similarly, miR‐23a‐5p can interact with the discovered serum mRNA profiles to regulate the AKT/mTOR pathway and can target molecules that are also targets of drugs, including capivasertib.

## 4 DISCUSSION

Here we presented novel findings from smRNA‐seq and whole‐transcriptome analyses in paired saliva and serum samples from OSCC patients and healthy individuals of promising molecular biomarkers for further validation using non‐invasive methods. We showed that more than 1300 miRNAs can be identified in the saliva and serum of OSCC, and healthy individuals, and present 12 novel miRNAs whose biological role would be further identified. We also demonstrated a set of seven unique saliva and serum miRNA and mRNA signatures, associated with OSCC, especially from patients with a smoking history, that were not identified in previous explorations by using microarray assays.^12^, ^13^, ^14^, ^15^, ^16^ We showed the predicted interactions of these miRNAs through canonical pathways, such as PI3K/AKT/mTOR pathway, and targets that can be also targeted by known drugs. Specifically, we presented, for the first time, that using paired saliva and serum from the same individuals and deep sequencing analyses, we can provide combined miRNA and mRNA signatures associated with OSCC and canonical pathways that may be useful for clinical testing. Although further validation in an adequate cohort of samples is needed to verify the diagnostic advantage of the combined panel of saliva and serum molecular biomarkers compared to analyses of either saliva or serum alone, we believe that based on our data, serum may provide highly sensitive biomarkers, and saliva from the same individual includes very specific biomarkers for the disease, so their combination can be a valuable tool for further clinical testing.

We also presented the association of a set of saliva and serum miRNAs and mRNAs with a history of tobacco smoking. In addition, we discovered 12 novel miRNAs in the extracellular fluid of both OSCC patients and healthy individuals, encouraging further analysis to reveal their biological role in cancer development and progression.

Specifically, we showed a novel panel of five miRNAs—saliva hsa‐miR‐7704 and hsa‐miR‐3648‐5p and serum hsa‐miR‐23a‐5p, hsa‐miR‐499a‐5p, and hsa‐miR‐556‐5p—to be the most significantly altered in OSCC patients relative to healthy individuals and associated with cancer‐related canonical pathways. The differential expression of miR‐7704, miR‐3648‐p, and miR‐556‐5p has been previously reported in the serum of patients with ovarian, bladder, and prostate cancer, respectively.^29^, ^30^, ^31^ One single prior study, including data from qPCR analysis of a panel of miRNAs, showed reduced levels of miR‐23a in the serum of patients with benign salivary gland neoplasms compared to controls.^32^ Here, we suggest serum hsa‐miR‐449a‐5p and hsa‐miR‐23a‐5p showed the highest levels of sensitivity, and saliva hsa‐miR‐7704 and hsa‐miR‐3648‐5p showed the highest levels of specificity, for further verification in an independent cohort of patients. Specifically, we suggest for the first time, miR‐7704 significant upregulation, as a potential index for OSCC in saliva. We also showed the significant downregulation of saliva miR‐187‐3p in OSCC smokers, and suggest further experimental studies to verify this association.

We also showed for the first time by whole‐transcriptome analysis in paired saliva and serum unique mRNA profiles associated with OSCC that overlap HNC‐related genes in the dataset and we showed that among 912 significantly differentially expressed genes in the saliva and serum of OSCC patients compared to healthy individuals, 64 are significantly altered genes, and 16 genes of them—*TNC*, *MMP‐10*, *TP63*, *RELA*(*p65*), *TCAIM*, *PTGS2*, *CXCL1*, *MPO*, *BAX*, *TLR2*, *RACGAP1*, *ITBG4*, *KDR*, *CAMK2D*, *TCAIM*, and *BRCA1*—are annotated with relevant biological roles in cancer‐related pathways, such as tumor microenvironment pathways, IL‐8 signaling, NF‐κB signaling etc., and can target other molecules that are also targets of drugs, as discussed below. We showed that the top five of them—*TNC*, *MMP‐10*, *TP63*, *RELA* (*p65*), and *TCAIM*—exhibited biological significance in the saliva and serum of OSCC relative to healthy individuals. Finally, we suggest three of them*—TNC*, *RELA* (*p65*), and *TCAIM*, with the highest levels of specificity (100%). Because *RELA* (*p65*) is a central factor in cancer that based on our prior preclinical and clinical data plays an important role in the progression of HNC,^1^, ^3^, ^4^, ^43^ and according to our data presented here, *RELA*(*p65*) was found one of the most altered genes in serum of OSCC versus HC (>25 fold changes, *p* < 0.05), we selected this gene to verify our sequencing data by qPCR and IHC in tumor tissue. Our observations from qPCR analysis verified the elevated *RELA* (p65) mRNAs in the serum of OSCC patients. In addition, the strong immunoreaction of NF‐κB in the tumor tissue verifies the activation of NF‐κB in the tumor cells relative to their ANTs which may reflect the molecular oncogenic alterations in both the tumor microenvironment and tumor secretions. Based on our current data, further testing of *TNC*, *RELA* (*p65*), and *TCAIM* molecules in an independent cohort of patients is merited to validate their diagnostic role in OSCC.

In addition, we presented data from target prediction mRNA analysis, that showed that mRNA profiles of oral cancer secretions or circulation, such as *TNC* (tenascin‐C) and *MMP‐10* in saliva, and *PTGS2* and *RELA* in serum, are linked to oncogenic function through inflammatory and cancer‐related pathways, such as IL‐8 and PI3K/AKT and tumor microenvironment pathways, targeting molecules that are also targets of drugs, suggesting their potential use as an index of treatment monitoring. The role of serum RNAs as novel potential biomarkers and therapeutic targets for oral cancer has been discussed in a recent article.^44^ In addition, based on our prior in vivo studies, these genes are progressively significantly deregulated during head and neck carcinogenesis,^1^, ^2^, ^3^, ^4^ supporting their further investigation in monitoring malignant development and progression in the oral cavity.

Finally, we present data from pathway analyses that showed that saliva and serum miRNAs can interact with mRNA profiles through the PI3K/AKT pathway, as well as their predicted targets, and suggested miR‐7704, miR‐1273 h‐5p, miR‐1180‐3p, miR‐499‐5p, and miR‐23a‐5p, for future therapeutic explorations or candidate biomarkers of the effectiveness of targeted therapy for oral cancer. Associations between long non‐coding RNAs in the serum of metastatic OSCC with PI3K/AKT have been previously suggested.^45^ PI3K/AKT/mTOR signaling is active in over 90% of HNSCC and is well‐known to play a role in HNC^39^ as well as a potential target for new therapy options. Inhibitors against PI3K, AKT, and mammalian target of rapamycin (mTOR) have remarkable effects on tumor cell proliferation and radiotherapy sensitization in cell cultures and mouse models. Based on our data the discovered miRNA panel merits further investigation to clarify their potential use in therapeutic applications or candidate biomarkers of the effectiveness of targeted therapy through the PI3K/AKT pathway. In addition, recent data support the role of exosomal miR‐7704 from mesenchymal stromal cells, as influential modulators of macrophage polarization,^46^ suggesting further investigation of miR‐7704 role in the stroma microenvironment of OSCC.

In conclusion, by using paired saliva and serum samples from OSCC patients and healthy individuals and deep sequencing analyses we discovered 12 novel miRNAs and a unique signature of combined saliva and serum miRNAs and mRNAs associated with OSCC may be useful for clinical testing to monitor patients, contributing to effective preventive care. We also provided novel data that both saliva and serum miRNAs can interact with mRNA factors through the PI3K/AKT/mTOR pathway in oral cavity cancer, supporting their further evaluation as potential biomarkers or targets for oral cancer therapy.

## AUTHOR CONTRIBUTIONS

**Dimitra P. Vageli:** Conceptualization (lead); data curation (lead); formal analysis (lead); investigation (lead); methodology (lead); project administration (lead); resources (equal); software (lead); supervision (lead); validation (lead); visualization (lead); writing – original draft (lead); writing – review and editing (lead). **Panagiotis G. Doukas:** Conceptualization (equal); data curation (equal); formal analysis (equal); investigation (equal); methodology (equal); software (equal); validation (equal); writing – review and editing (equal). **Jeffrey P. Townsend:** Validation (equal); writing – review and editing (equal). **Curtis Pickering:** Validation (equal); visualization (equal); writing – review and editing (equal). **Benjamin L. Judson:** Conceptualization (equal); data curation (equal); funding acquisition (lead); investigation (equal); project administration (equal); resources (equal); supervision (equal); validation (equal); writing – review and editing (equal).

## CONFLICT OF INTEREST STATEMENT

The authors declare that no conflict could be perceived as prejudicing the impartiality of the research.

## ETHICS STATEMENT

All participants provided oral and written consent for participation in this study and donation of their biomedical samples to the Yale Head and Neck Biorepository (HIC#1206010419). All participants agreed to the publication of the information presented in the current study and all identifying information was removed.

## Supporting information


Data S1.

## Data Availability

The data that support the findings of this study are available in the supplementary section and upon request to the corresponding author. .
