# Supplementary material for: Novel non‐invasive molecular signatures for oral cavity cancer, by whole transcriptome and small non‐coding RNA sequencing analyses: Predicted association with PI3K/AKT/mTOR pathway
Source: Cancer Med. 2024 May 31;13(11):e7309. doi: 10.1002/cam4.7309 (PMC11141334; doi:10.1002/cam4.7309)
Supplement: Supplementary file 1 — Data S1. [file CAM4-13-e7309-s001.docx]

**Novel non-invasive molecular signatures for oral cavity cancer, by whole transcriptome and small non-coding RNA sequencing analyses: predicted association with PI3K/AKT/mTOR pathway.**

^1^Dimitra P. Vageli, PhD; ^1,2^Panagiotis G. Doukas, MD; ^3^Jeffrey P. Townsend, PhD, ^4^Curtis Pickering, PhD and ^1^Benjamin L. Judson, MD

^1^Yale Larynx Lab, Surgery Otolaryngology; Yale Medical School; 310 Cedar Street (BML 212); New Haven, CT 06510; ^2^Department of Medicine Saint Peter's University Hospital/Rutgers- RWJ Medical School, 254 Easton Avenue, New Brunswick, NJ, 08901; ^3^Department of Biostatistics, Yale School of Public Health; ^4^Department of Surgery; Division of Otolaryngology; Yale Medical School, New Haven CT 06510, USA.

Corresponding author: Dimitra Vageli, PhD; Yale Larynx lab, Surgery Otolaryngology; Yale Medical School; 310 Cedar Street (BML 212); New Haven, CT 06510, USA.

e-mail: [dimitra.vangeli@yale.edu](mailto:dimitra.vangeli@yale.edu)

Office: 203-737-1447

**Supplementary methods**

*Sequencing analyses of whole transcriptome and small non-coding RNA:* Total RNA was isolated from whole saliva and serum using miRNeasy and mRNAeasy Serum/Plasma Advanced Kits (Qiagen Inc.), respectively, following the manufacturer’s instructions, as described in supplementary data. The quality and quantity of total RNA was determined by absorption ratios at 260/280 nm (≥2.0) and concentration ratios by absorption at 260 nm, using a NanoDrop^TM^ 1000 spectrophotometer (Thermo Scientific). Total RNA quality was determined by estimating the A260/A280 and A260/A230 ratios by nanodrop. RNA integrity was determined by running an Agilent Bioanalyzer gel, which measures the ratio of the ribosomal peaks. Samples with RIN values of 7 or greater were recommended for library prep.

(i) *RNA Seq Library Prep (*Roche Kapa mRNA Hyper Prep, Cat# KR1352): indexed libraries were quantified by qRT-PCR using Roch KAPA Biosystems, Cat# KK4854, and insert size distribution was determined by the Agilent Bioanalyzer. Samples with a yield of ≥ 0.5 ng/μl and a size distribution of 150-300 bp were used for sequencing.

*(ii) mRNA-seq:* We trimmed low-quality reads and removed adaptor contamination using Trim Galore (v0.5.0) before mRNA-seq data analysis. Subsequently, we mapped the trimmed reads to the human reference genome (hg38) using HISAT2 (v2.1.0). We quantified the gene expression levels using StringTie (v1.3.3b), with gene models (v27) from the GENCODE project.

(iii) *miRNA Seq Library Prep:* (Qiagen miRNA Library Prep Kit, Cat# 331505): the library run on the Agilent Tapestation. The library was quantified by qRT-PCR using KAPA Biosystems kit. Sample concentrations were normalized to 1.2 nM and loaded onto an Illumina NovaSeq flow cell at a concentration that yields 15-25 million passing filter clusters per sample. Samples were sequenced using 100bp paired-end sequencing according to Illumina protocols. Data generated during sequencing runs was simultaneously transferred to the YCGA high-performance computing cluster and stored. A positive control provided by Illumina was spiked into every lane at a concentration of 0.3% to monitor sequencing quality in real-time.

*(iv) miRNA-seq:* We used the miRge3.0 pipeline to align the raw reads against the human small-RNA annotation library (SourceForge; CA. USA) and identify novel miRNAs in deep sequencing data.

We identified the top 32 miRNA and mRNA genes (up and downregulated) based on the highest levels of differential expression (log_2_ fold-change and *P*_adj_ values by DESeq2; False-discovery rate ≤ 0.05). Then we identified their biological role, as described below. We assessed statistical significance of differential expression between OSCC versus HC by *t*-test (*P* < 0.05).

*Reverse transcription and qPCR:* We used 30 ng of each serum RNA sample and performed reverse transcription to cDNA using iScript cDNA synthesis kit (Qiagen®, KY, USA), following the manufacturer’s instructions. We diluted 1:10 of cDNA in nuclease-free water and performed qPCR to determine the differential mRNA expression of *RELA(p65)* in serum from OSCC patients compared with HC. We used a Bio-Rad real-time thermal cycler (Bio-Rad real-time thermal cycler CFX96^TM^; Bio-Rad, Hercules, CA, USA) and specific primers for the human genome (QuantiTect® primers assay, Qiagen®, KY, USA; *hGAPDH:* NM_001256799, NM_002046 (95 bp); *RELA:* NM_001145138, NM_001243984-5, NM_021975 (107 bp)] and iQ^TM^ SYBR® Green Supermix (BIO-RAD, CA, USA). We used *hGAPDH* gene as a reference control gene and normalized expression ratios for the *target gene/hGADGH* were estimated by CFX96^TM^ software (Bio-Rad, CA, USA), for all serum samples. qPCR assays were performed in 96-well plates and each sample was assayed in triplicate.

*Immunohistochemical analysis:* We used chromogenic staining in tissue sections of 4-5 µm from representative OSCC specimens. Positive and non-template negative controls were used in each IHC assay as recommended by the manufacturer. Slides were stained with primary mouse monoclonal antibodies for NF-*κ*B p65 (F-6) (*Santa Cruz Biotechnology., Inc.*, Europe), followed by secondary antibody conjugated to Horseradish Peroxidase (HRP) (m-IgG_1_ BP-HRP; *Santa Cruz Biotechnology., Inc.*, Europe); and peroxidase substrate (3-3′ diaminobenzidine tetrachloride, DAB; *Santa Cruz Biotechnology., Inc.*, Europe). Subsequently, slides were examined, and at least three images per tissue section were captured and analyzed using a light Leica microscope, Aperio CS2 system, and Image Scope software (Leica microsystems, IL, USA).

**Supplementary Table S1**: Clinical, histological, and social history characteristics of study (OSCC) and control (HC) participants.

| **OSCC** | **Sex** | **Age (years)** | **Tobacco use, (py)** | **Alcohol use** | **GERD** | **Histology** | **Tumor grade (differentiation**) | **Tumor stage (TNM) /distal metastasis** |
| --- | --- | --- | --- | --- | --- | --- | --- | --- |
| NS | M | 54 | NS | Yes | No | SCC | Moderate | T4N1aMx / Yes |
|  | F | 84 | NS | Yes | No | SCC | Well | T1N1Mx |
|  | M | 61 | NS | No | Yes | SCC | Well | T3N0M0 |
|  | F | 79 | NS | Yes | Yes | SCC | Moderate | T2N1M0 |
| SM | F | 62 | SM (former), 30py | Yes | No | SCC | Well | T3N0M0 |
|  | M | 80 | SM, 40py | No | No | SCC | Poor | T1N1Mx / Yes |
|  | M | 70 | SM (former), 30py | No | No | SCC | Well | T2N0M0 |
|  | F | 80 | SM (former), 5py | Yes | No | SCC | N/A | T2N0M0 |

| **HC** | **Sex** | **Age**  **(years)** | **Tobacco use, (py)** | **Alcohol use** | **GERD** | **Histology** |
| --- | --- | --- | --- | --- | --- | --- |
| NS | F | 67 | NS | No | No | N/A |
|  | M | 82 | NS | No | No | Zenker's diverticulum |
|  | M | 76 | NS | No | No | Zenker's diverticulum |
|  | F | 59 | NS | No | Yes | Neck mass |
| SM | M | 36 | SM, 30py | No | Yes | Branchial neck cyst |
|  | M | 38 | SM (former), 3.75py | No | No | Eagle's syndrome |
|  | M | 54 | SM (former), 10py | No | No | Neck benign lipoma |
|  | F | 64 | SM (former), 5py | Yes | Yes | Hypertrophic tonsil |

NS: never smokers; SM: Smokers; M: male; F: Female; PY: pack year; SCC: squamous cell carcinoma; N/A: non-applied

**Supplementary Table S2:** Calculation of individual sensitivity and positivity of the top 10 saliva and serum miRNA and mRNAs for OSCC.

| miRNAs and mRNAs – secretion of OSCC (Positive or negative detection) | a=True positive, b=False positive, c=False negative, d=True negative | *Sensitivity; **Specificity |
| --- | --- | --- |
| hsa-miR-499a-5p serum (positive) | a=7, b=6, c=1, d=2 | 0.875; 0.25 |
| MMP-10- saliva (positive) | a=6, b=2, c=2, d=6 | 0.75; 0.75 |
| TP63 – saliva (positive) | a=6, b=1, c=2, d=7 | 0.75; 0.875 |
| hsa-miR-556-5p – serum (negative) | a=6, b=0, c=2, d=8 | 0.75; 1 |
| hsa-miR-23a-5p – serum (negative) | a=6, b=0, c=2, d=8 | 0.75; 1 |
| TNC – saliva (positive) | a=5, b=0, c=3, d=8 | 0.625; 1 |
| hsa-miR-7704 – saliva (positive) | a=3, b=0, c=5, d=8 | 0.375; 1 |
| hsa-miR-3648-5p – saliva (positive | a=3, b=0, c=5, d=8 | 0.375; 1 |
| RELA – serum (positive) | a=3, b=0, c=3, d=8 | 0.50; 1 |
| TCAM1 -serum (positive) | a=4, b=0, c=4, d=8 | 0.50; 1 |

***** Sensitivity: a / (a + c); **Specificity: d / (b + d)

**Supplementary Table S3: Saliva broad molecular analysis for miRNAs in OSCC.**

| *Study | Subjects (analyzed miRNAs) / analysis | Saliva | Tissue | Serum | Differentially expressed miRNAs/Biomarkers |
| --- | --- | --- | --- | --- | --- |
| Our study **2023** | 8 OSCC + 8 HC (**2731** miRNAs) / smRNA-seq | Yes |  | Yes | 22 miRNAs **/miR-7704, miR-3648, miR-1293-5p, miR-3611-3p, miR-499a-5p, miR-23a-5p, miR-556-5p** |
| ^19^Romani C, **2021** | 50 OSCC + 42 HC (**1361** miRNAs) / microarray | Yes | Yes |  | 25 miRNAs/**miR-423-5p** |
| ^18^Duz MB, **2016** | 3 OSCC + 4 HC (**2006** miRNAs) / microarray | Yes |  |  | 419 miRNAs/**miR-139-5p** |
| ^17^Momen-Heravi F, **2014** | 17 OSCC + 9 HC (**734** miRNAs) /NanoString nCounter | Yes |  |  | 13 miRNAs/**miRNA-136, miR-27b** |
| ^16^Salazar C, **2014** | 5 HNSCC + 5 HC (**84** miRNAs) / microarray | Yes |  |  | 5 miRNAs/**miR-134, miR-9, miR-191** |
| ^15^Park NJ, **2009** | 12 OSCC + 12 HC (**319** miRNAs) / qPCR | Yes |  |  | 28 miRNAs/**miR-200a, miR-125a** |

*Reference; HNSCC: Head and neck Squamous Cell Carcinoma; OSCC: Oral Squamous Cell Carcinoma; HC: Healthy Control

**Supplementary Table S4: A.** Differentially expressed miRNAs in saliva and serum of OSCC vs. HC. **B.** Significantly altered miRNAs in saliva and serum of OSCC vs. HC, smokers and never smokers.

**A.**

| **GENES** | **DIFFERENTIAL EPRESSION** | |
| --- | --- | --- |
| **SALIVA OSCC vs HC** | **log2FoldChange** | ***P* value** |
| hsa-miR-3648-5p | 24.6 | 2.2285E-16 |
| hsa-miR-7704 | 25.2 | 5.6550E-15 |
| hsa-miR-1180-3p | 21.4 | 6.8387E-11 |
| hsa-miR-1273h-5p | 20.9 | 2.6701E-10 |
| hsa-miR-196a-5p | 7.7 | 1.807E-03 |
| hsa-miR-421 | -0.7 | 4.059E-03 |
| hsa-miR-339-3p | 0.7 | 5.029E-03 |
| hsa-miR-1246 | 1.4 | 6.908E-03 |
| hsa-miR-181b-5p | 0.8 | 2.223E-02 |
| hsa-miR-192-5p/215-5p | -1.1 | 2.444E-02 |
| hsa-miR-362-3p | -6.2 | 2.464E-02 |
| hsa-miR-3196 | 6.7 | 2.478E-02 |
| hsa-miR-30b-5p/30c-5p | -0.6 | 2.550E-02 |
| hsa-miR-23a-3p/23b-3p | -0.5 | 3.125E-02 |
| hsa-miR-7706 | 6.4 | 3.146E-02 |
| hsa-miR-345-5p | 0.6 | 3.702E-02 |
| hsa-miR-128-3p | 0.5 | 3.712E-02 |
| hsa-miR-92a-1-5p | -6.1 | 4.016E-02 |
| hsa-miR-151a-3p | 0.8 | 4.179E-02 |
| hsa-miR-4508 | 6.0 | 4.351E-02 |
| hsa-miR-660-3p | -5.6 | 4.781E-02 |
| hsa-miR-224-5p | 0.8 | 4.888E-02 |
| **SERUM OSCC vs HC** | **log2FoldChange** | ***P* value** |
| hsa-miR-23a-5p | -6.5 | 3.584E-07 |
| hsa-miR-499a-5p | 2.6 | 1.183E-05 |
| hsa-miR-556-5p | -5.3 | 6.752E-05 |
| hsa-miR-202-3p | -4.6 | 3.852E-04 |
| hsa-miR-12136 | 2.6 | 4.334E-04 |
| hsa-miR-625-5p | 0.9 | 1.189E-03 |
| hsa-miR-616-3p | -5.1 | 1.451E-03 |
| hsa-miR-6724-5p | -5.4 | 1.751E-03 |
| hsa-miR-4492 | 1.6 | 2.763E-03 |
| hsa-miR-5187-5p | -5.9 | 2.975E-03 |
| hsa-miR-1246 | 2.1 | 6.749E-03 |
| hsa-miR-6803-5p | 7.4 | 7.321E-03 |
| hsa-miR-1249-5p | 7.8 | 8.911E-03 |
| hsa-miR-1250-5p | -4.1 | 1.183E-02 |
| hsa-miR-340-5p | 0.9 | 1.228E-02 |
| hsa-miR-190b-5p | -4.8 | 1.414E-02 |
| hsa-miR-25-5p | 0.8 | 1.514E-02 |
| hsa-miR-6850-5p | -4.8 | 1.515E-02 |
| hsa-miR-138-5p | -2.9 | 1.651E-02 |
| hsa-miR-190a-5p | -3.9 | 1.807E-02 |
| hsa-miR-3138-3p | -5.5 | 1.956E-02 |
| hsa-miR-1260a/1260b | -5.5 | 2.095E-02 |
| hsa-miR-181c-5p | 1.5 | 2.223E-02 |
| hsa-miR-651-5p | 1.2 | 2.231E-02 |
| hsa-miR-187-3p | -4.6 | 2.816E-02 |
| hsa-miR-205-5p | 1.3 | 2.836E-02 |
| hsa-miR-3127-5p | -4.3 | 3.293E-02 |
| hsa-miR-10396a-5p/10396b-5p | -4.6 | 3.354E-02 |
| hsa-miR-324-5p | -0.6 | 3.432E-02 |
| hsa-miR-6884-5p | -4.3 | 3.433E-02 |
| hsa-miR-873-5p | -3.9 | 3.464E-02 |
| hsa-miR-3691-3p | 6.2 | 3.577E-02 |
| hsa-miR-3177-3p | 3.2 | 3.631E-02 |
| hsa-miR-654-3p | -2.7 | 3.708E-02 |
| hsa-miR-4804-5p | -4.2 | 3.721E-02 |
| hsa-miR-4467-5p | -3.7 | 3.757E-02 |
| hsa-miR-32-3p | 0.9 | 3.876E-02 |
| hsa-miR-1306-3p | -2.6 | 3.896E-02 |
| hsa-miR-6515-5p | -5.0 | 3.898E-02 |
| hsa-miR-574-5p | 0.9 | 4.267E-02 |
| hsa-miR-339-3p | -1.0 | 4.512E-02 |
| hsa-miR-641 | -4.1 | 4.696E-02 |
| hsa-miR-34a-5p | 1.1 | 4.843E-02 |

| **B.**  **GENES** | **DIFFERENTIAL EPRESSION** | |
| --- | --- | --- |
| **SALIVA OSCC NS vs HC NS** | **log2FoldChange** | **padj** |
| hsa-miR-1246 | 2.0 | 4.1816E-02 |
| hsa-miR-1271-5p | -6.1 | 1.5923E-02 |
| hsa-miR-1293-5p | 21.7 | 1.9047E-05 |
| hsa-miR-141-5p | -7.9 | 8.9630E-03 |
| hsa-miR-2115-5p | 20.3 | 8.6916E-05 |
| hsa-miR-3611-3p | -20.9 | 1.9047E-05 |
| hsa-miR-3648-5p | 10.7 | 2.8381E-02 |
| hsa-miR-4488 | 2.8 | 2.8970E-04 |
| hsa-miR-4497 | 10.0 | 4.1284E-03 |
| hsa-miR-4521 | -20.5 | 1.9047E-05 |
| hsa-miR-455-5p | -20.5 | 1.9047E-05 |
| hsa-miR-486-5p | 5.2 | 1.5923E-02 |
| hsa-miR-556-5p | -21.5 | 3.8090E-12 |
| hsa-miR-582-3p | -20.6 | 8.8493E-12 |
| **SALIVA OSCC SM vs HC SM** | **log2FoldChange** | **padj** |
| hsa-miR-1293-5p | -35.9 | 6.3634E-15 |
| hsa-miR-187-3p | -23.2 | 7.5182E-06 |
| **SALIVA OSCC SM vs OSCC NS** | **log2FoldChange** | **padj** |
| hsa-miR-1246 | -2.2 | 1.0658E-02 |
| hsa-miR-1293-5p | -38.0 | 6.0913E-17 |
| hsa-miR-141-5p | 7.6 | 1.0658E-02 |
| hsa-miR-187-3p | -26.1 | 4.7423E-08 |
| hsa-miR-324-3p | -24.9 | 2.3629E-07 |
| hsa-miR-3611-3p | 20.8 | 7.4399E-06 |
| hsa-miR-3648-5p | -11.0 | 1.6747E-02 |
| hsa-miR-4449 | -10.3 | 9.6126E-03 |
| hsa-miR-4488 | -2.4 | 4.2821E-03 |
| hsa-miR-4521 | 21.3 | 3.9259E-06 |
| hsa-miR-455-5p | 21.3 | 3.9259E-06 |
| hsa-miR-556-5p | 22.4 | 8.5038E-14 |
| hsa-miR-582-3p | 22.2 | 8.2575E-14 |
| **SERUM OSCC SM vs HC SM** | **log2FoldChange** | **padj** |
| hsa-miR-499a-5p | 3.3 | 1.7697E-02 |
| hsa-miR-885-3p | -23.0 | 8.6367E-21 |
| **SERUM OSCC SM vs OSCC NS** | **log2FoldChange** | **padj** |
| hsa-miR-885-3p | -21.7 | 3.4484E-18 |

**Supplementary Table S5:** Novel miRNAs identified by smRNA-seq in saliva or serum of OSCC or HC. Novel miRNAs in saliva or serum of both OSCC and HC

|  | **SALIVA NOVEL miRNAs** | | | | | | |
| --- | --- | --- | --- | --- | --- | --- | --- |
| **ID** | **Probability** | **Chr** | **Start pos.** | **End Pos.** | **Mature miRNA sequence** | **Read count** |  |
| 1 | 0.921756 | chr1 | 228647814 | 228647831 | UGUGGGCUUGGGCGUGUG | 16 | |
| 2 | 0.814690 | chr17 | 46771080 | 46771096 | UGCUGCAAGAAGGAAGC | 17 | |
| 3 | 0.912159 | chr13 | 74065559 | 74065578 | UGAGAAUAUGAGUGUUUGUG | 213 | |
| 4 | 0.840098 | chr10 | 69315231 | 69315246 | UCAGAGUGUGCUCCCA | 36 | |
| 5 | 0.994683 | chr2 | 22542187 | 22542203 | UAAGAAUGGAAUGUAGU | 10 | |
| 6 | 0.899305 | chr9 | 85986499 | 85986511 | GUGGAAGUCAGAG | 20 | |
| 7 | 0.972849 | chrUn_GL000220v1 | 118137 | 118156 | GCGGGCCGCCCCCCCCCUCC | 21 | |
| 8 | 0.972849 | chr22_KI270733v1_random | 135000 | 135019 | GCGGGCCGCCCCCCCCCUCC | 21 | |
| 9 | 0.972849 | chr21 | 8401700 | 8401719 | GCGGGCCGCCCCCCCCCUCC | 21 | |
| 10 | 0.811461 | chr6 | 6826109 | 6826121 | GAGCUGGGGCAUA | 14 | |
| 11 | 0.916363 | chr10 | 96136369 | 96136381 | GAAAGGUAAGAAG | 18 | |
| 12 | 0.999999 | chr22 | 50191179 | 50191198 | CGGCUGUCCAAGAAGAGGGC | 35 | |
| 13 | 0.990177 | chr22 | 50191179 | 50191196 | CGGCUGUCCAAGAAGAGG | 13 | |
| 14 | 0.999999 | chr3 | 52393993 | 52394013 | CCUUCUCGAGCCUUGAGUGUG | 46 | |
| 15 | 0.989648 | chr22 | 50191178 | 50191197 | CCGGCUGUCCAAGAAGAGGG | 11 | |
| 16 | 0.968595 | chr22 | 50191178 | 50191196 | CCGGCUGUCCAAGAAGAGG | 18 | |
| 17 | 0.985129 | chr3 | 52394001 | 52394012 | AGCCUUGAGUGU | 39 | |
| **SERUM NOVEL miRNAs** | | | | | | | |
| **ID** | **Probability** | **Chr** | **Start pos.** | **End Pos.** | **Mature miRNA sequence** | **Read count** |  |
| 1 | 0.999999 | chr1 | 26554542 | 26554561 | GCAGCAAGGAAGGCAGGGGU | 731 | |
| 2 | 0.999999 | chr8 | 1755133 | 1755153 | UUGCUGGGAAAGGGAGAAGUU | 14 | |
| 3 | 0.999999 | chr11 | 12321513 | 12321533 | GCAGGAAGAGACGCAGCAGCU | 13 | |
| 4 | 0.999997 | chr2 | 111135385 | 111135405 | UUGUGUCCAGUUGUUGGGGGA | 59 | |
| 5 | 0.999984 | chr8 | 1755134 | 1755152 | UGCUGGGAAAGGGAGAAGU | 15 | |
| 6 | 0.980306 | chr17 | 44219190 | 44219211 | CGGCCGGGGAGAACUGCGCCUG | 12 | |
| 7 | 0.985564 | chr6 | 119823108 | 119823128 | UGAGCAAGUGAAGUAUGUGGU | 54 | |
| 8 | 0.970637 | chr17 | 17836469 | 17836487 | UGUCCCGGCCCGGAGCCGC | 13 | |
| 9 | 0.967191 | chr1 | 26554543 | 26554561 | CAGCAAGGAAGGCAGGGGU | 55 | |
| 10 | 0.943951 | chr12 | 121803498 | 121803519 | CGGGCCGCAGGAGGAAGCCGGA | 26 | |
| 11 | 0.9290604 | chr22 | 50191178 | 50191196 | CCGGCUGUCCAAGAAGAGG | 11 | |
| 12 | 0.928911 | chr7 | 142474569 | 142474589 | CACAGUGUGGCACAGUCGUGU | 24 | |
| 13 | 0.926711 | chr11 | 117181972 | 117181994 | GUCUGCAGGGUAGAGUGAGGGGA | 18 | |
| 14 | 0.911615 | chr17 | 17694939 | 17694957 | AGGCGGCGCGCAUCUCUGG | 18 | |
| 15 | 0.902322 | chr3 | 52057560 | 52057580 | CAGGCGGGGCAGCGGAGGUGA | 15 | |
| 16 | 0.890503 | chr7 | 99391118 | 99391137 | CAGGGGAGGAGGUGAGUGCA | 13 | |
| 17 | 0.883668 | chr8 | 98294205 | 98294224 | GAGGAGGCCGCGCCGCAGCC | 11 | |
| 18 | 0.818921 | chr2 | 180678114 | 180678131 | AAGGCGGGGUGUGGGAGA | 12 | |
| 19 | 0.817921 | chr13 | 24923174 | 24923192 | AGCAGGGAGCACGGAGAGU | 32 | |
| 20 | 0.813981 | chr15 | 40807130 | 40807151 | CCACGGGGAGAGCACAGCCACC | 13 | |

Red: Novel miRNAs expressed in saliva or serum of both OSCC patients and healthy individuals.

**Supplementary Table S6:** Downstream effects analysis by IPA provided evidence of OSCC-associated saliva (A) and serum (B) genes overlapping Head and Neck Cancer genes in the dataset and their predicted effects.

| © 2000-2023 QIAGEN. All rights reserved. | | |  |  |
| --- | --- | --- | --- | --- |
| ID | Genes in dataset | Prediction (based on measurement direction) | Expr Log Ratio | Findings |
| ENSG00000153303 | FRMD1 | Affected | 23.714 | Affects (5) |
| ENSG00000153993 | SEMA3D | Affected | 9.252 | Affects (4) |
| ENSG00000241186 | TDGF1 | Increased | 9.057 | Increases (4) |
| ENSG00000164741 | DLC1 | Affected | 8.457 | Affects (3) |
| ENSG00000044524 | EPHA3 | Affected | 8.158 | Affects (3) |
| ENSG00000109193 | SULT1E1 | Affected | 8.135 | Affects (1) |
| ENSG00000182168 | UNC5C | Affected | 8.016 | Affects (8) |
| ENSG00000041982 | TNC | Affected | 8.012 | Affects (11) |
| ENSG00000165923 | AGBL2 | Affected | 7.931 | Affects (5) |
| ENSG00000091137 | SLC26A4 | Affected | 7.831 | Affects (5) |
| ENSG00000139910 | NOVA1 | Affected | 7.708 | Affects (17) |
| ENSG00000166263 | STXBP4 | Affected | 7.403 | Affects (3) |
| ENSG00000165061 | ZMAT4 | Affected | 7.344 | Affects (1) |
| ENSG00000152894 | PTPRK | Affected | 7.27 | Affects (5) |
| ENSG00000135097 | MSI1 | Affected | 7.253 | Affects (4) |
| ENSG00000151773 | CCDC122 | Affected | 6.996 | Affects (1) |
| ENSG00000107186 | MPDZ | Affected | 6.979 | Affects (10) |
| ENSG00000222036 | POTEM (includes others) | Affected | 6.957 | Affects (22) |
| ENSG00000152527 | PLEKHH2 | Affected | 6.917 | Affects (7) |
| ENSG00000198453 | ZNF568 | Affected | 6.867 | Affects (1) |
| ENSG00000164778 | EN2 | Affected | 6.86 | Affects (5) |
| ENSG00000109819 | PPARGC1A | Affected | 6.825 | Affects (4) |
| ENSG00000039139 | DNAH5 | Affected | 6.805 | Affects (35) |
| ENSG00000166670 | MMP10 | Affected | 6.776 | Affects (4) |
| ENSG00000036565 | SLC18A1 | Affected | 6.696 | Affects (2) |
| ENSG00000179271 | GADD45GIP1 | Affected | 6.687 | Affects (3) |
| ENSG00000120075 | HOXB5 | Affected | 6.546 | Affects (1) |
| ENSG00000165186 | PTCHD1 | Affected | 6.508 | Affects (5) |
| ENSG00000204186 | ZDBF2 | Affected | 6.323 | Affects (7) |
| ENSG00000177707 | NECTIN3 | Affected | 6.315 | Affects (1) |
| ENSG00000198816 | ZNF358 | Affected | 6.3 | Affects (8) |
| ENSG00000102781 | KATNAL1 | Affected | 6.288 | Affects (1) |
| ENSG00000040731 | CDH10 | Affected | 6.233 | Affects (3) |
| ENSG00000112182 | BACH2 | Affected | 6.109 | Affects (3) |
| ENSG00000179909 | ZNF154 | Affected | 5.995 | Affects (2) |
| ENSG00000181458 | TMEM45A | Affected | 5.902 | Affects (1) |
| ENSG00000137960 | GIPC2 | Affected | 5.893 | Affects (4) |
| ENSG00000166206 | GABRB3 | Affected | 5.867 | Affects (2) |
| ENSG00000163281 | GNPDA2 | Affected | 5.765 | Affects (3) |
| ENSG00000198832 | SELENOM | Affected | 5.756 | Affects (4) |
| ENSG00000171723 | GPHN | Affected | 5.713 | Affects (2) |
| ENSG00000197128 | ZNF772 | Affected | 5.577 | Affects (1) |
| ENSG00000151229 | SLC2A13 | Affected | 5.376 | Affects (5) |
| ENSG00000006377 | DLX6 | Affected | 5.29 | Affects (6) |
| ENSG00000133216 | EPHB2 | Affected | 5.226 | Affects (2) |
| ENSG00000122970 | IFT81 | Affected | 5.216 | Affects (5) |
| ENSG00000140945 | CDH13 | Affected | 5.013 | Affects (7) |
| ENSG00000185046 | ANKS1B | Affected | 5.01 | Affects (7) |
| ENSG00000166133 | RPUSD2 | Affected | 4.8 | Affects (1) |
| ENSG00000119888 | EPCAM | Affected | 4.765 | Affects (4) |

**A. SALIVA**

**B. SERUM**

| © 2000-2023 QIAGEN. All rights reserved. | | | |  |
| --- | --- | --- | --- | --- |
| ID | Genes in dataset | Prediction (based on measurement direction) | Expr Log Ratio | Findings |
| ENSG00000132376 | INPP5K | Affected | 27.832 | Affects (4) |
| ENSG00000061936 | SFSWAP | Affected | 27.461 | Affects (6) |
| ENSG00000135919 | SERPINE2 | Affected | 27.351 | Affects (4) |
| ENSG00000161800 | RACGAP1 | Affected | 27.348 | Affects (2) |
| ENSG00000163510 | CWC22 | Affected | 27.234 | Affects (9) |
| ENSG00000111727 | HCFC2 | Affected | 27.129 | Affects (4) |
| ENSG00000145349 | CAMK2D | Affected | 26.959 | Affects (5) |
| ENSG00000149798 | CDC42EP2 | Affected | 26.933 | Affects (1) |
| ENSG00000197102 | DYNC1H1 | Affected | 26.912 | Affects (26) |
| ENSG00000179152 | TCAIM | Affected | 26.813 | Affects (3) |
| ENSG00000163687 | DNASE1L3 | Affected | 26.785 | Affects (6) |
| ENSG00000237441 | RGL2 | Affected | 26.768 | Affects (4) |
| ENSG00000102910 | LONP2 | Affected | 26.729 | Affects (6) |
| ENSG00000100227 | POLDIP3 | Affected | 26.574 | Affects (2) |
| ENSG00000157823 | AP3S2 | Affected | 26.526 | Affects (1) |
| ENSG00000118260 | CREB1 | Affected | 26.464 | Affects (1) |
| ENSG00000164120 | HPGD | Affected | 26.374 | Affects (4) |
| ENSG00000173039 | RELA | Affected | 26.332 | Affects (2) |
| ENSG00000088356 | PDRG1 | Affected | 26.241 | Affects (1) |
| ENSG00000101391 | CDK5RAP1 | Affected | 26.212 | Affects (8) |
| ENSG00000254858 | MPV17L2 | Affected | 26.2 | Affects (3) |
| ENSG00000073756 | PTGS2 | Affected | 26.167 | Affects (41) |
| ENSG00000110906 | KCTD10 | Affected | 26.148 | Affects (3) |
| ENSG00000159445 | THEM4 | Affected | 26.134 | Affects (3) |
| ENSG00000071794 | HLTF | Affected | 26.089 | Affects (7) |
| ENSG00000262664 | OVCA2 | Affected | 26.016 | Affects (2) |
| ENSG00000160219 | GAB3 | Affected | 25.802 | Affects (4) |
| ENSG00000171161 | ZNF672 | Affected | 25.731 | Affects (6) |
| ENSG00000168010 | ATG16L2 | Affected | 25.656 | Affects (9) |
| ENSG00000177853 | ZNF518A | Affected | 25.64 | Affects (4) |
| ENSG00000171456 | ASXL1 | Affected | 25.563 | Affects (13) |
| ENSG00000175893 | ZDHHC21 | Affected | 25.555 | Affects (5) |
| ENSG00000130150 | MOSPD2 | Affected | 25.494 | Affects (2) |
| ENSG00000120509 | PDZD11 | Affected | 25.419 | Affects (1) |
| ENSG00000136122 | BORA | Affected | 25.407 | Affects (3) |
| ENSG00000157954 | WIPI2 | Affected | 25.392 | Affects (4) |
| ENSG00000122515 | ZMIZ2 | Affected | 25.379 | Affects (6) |
| ENSG00000125734 | GPR108 | Affected | 25.336 | Affects (2) |
| ENSG00000132275 | RRP8 | Affected | 25.3 | Affects (1) |
| ENSG00000138686 | BBS7 | Affected | 25.275 | Affects (5) |
| ENSG00000014914 | MTMR11 | Affected | 25.254 | Affects (5) |
| ENSG00000014919 | COX15 | Affected | 25.254 | Affects (1) |
| ENSG00000182400 | TRAPPC6B | Affected | 25.231 | Affects (4) |
| ENSG00000163812 | ZDHHC3 | Affected | 25.223 | Affects (3) |
| ENSG00000176083 | ZNF683 | Affected | 25.209 | Affects (3) |
| ENSG00000112167 | SAYSD1 | Affected | 25.147 | Affects (1) |
| ENSG00000105499 | PLA2G4C | Affected | 25.131 | Affects (1) |
| ENSG00000141452 | RMC1 | Affected | 25.108 | Affects (4) |
| ENSG00000129158 | SERGEF | Affected | 25.107 | Affects (3) |
| ENSG00000063244 | U2AF2 | Affected | 25.03 | Affects (6) |

**Supplementary Table S7:** Saliva (A) and serum (B) mRNA profiles associated with OSCC.

**A.**

Top 32 highest overexpressed genes in the saliva of OSCC vs. HC.

| **SALIVA OSCC vs. HC** | | | |
| --- | --- | --- | --- |
| **GeneID** | **GeneName** | **log2FC** | **padj** |
| ENSG00000073282 | TP63 | 5.0 | 2.76050E-02 |
| ENSG00000166670 | MMP10 | 6.8 | 2.63150E-02 |
| ENSG00000240405 | SAMMSON | 7.3 | 1.49657E-02 |
| ENSG00000165061 | ZMAT4 | 7.3 | 1.73836E-02 |
| ENSG00000152894 | PTPRK | 7.3 | 3.16796E-02 |
| ENSG00000134874 | DZIP1 | 7.3 | 5.82177E-03 |
| ENSG00000140043 | PTGR2 | 7.4 | 3.70903E-02 |
| ENSG00000166263 | STXBP4 | 7.4 | 5.82177E-03 |
| ENSG00000205500 | AC013472.1 | 7.5 | 4.87012E-02 |
| ENSG00000127325 | BEST3 | 7.5 | 3.32366E-03 |
| ENSG00000260389 | WBP11P1 | 7.6 | 4.88361E-02 |
| ENSG00000261501 | AC079341.1 | 7.7 | 3.73354E-02 |
| ENSG00000139910 | NOVA1 | 7.7 | 3.32366E-03 |
| ENSG00000091137 | SLC26A4 | 7.8 | 3.70903E-02 |
| ENSG00000179136 | LINC00670 | 7.8 | 3.83781E-02 |
| ENSG00000165923 | AGBL2 | 7.9 | 9.93460E-03 |
| ENSG00000179577 | AP003471.1 | 7.9 | 2.41324E-02 |
| ENSG00000229961 | AL357143.1 | 7.9 | 1.26068E-03 |
| ENSG00000244128 | LINC01322 | 7.9 | 4.55722E-02 |
| ENSG00000182168 | UNC5C | 8.0 | 3.70903E-02 |
| ENSG00000041982 | TNC | 8.0 | 3.62815E-02 |
| ENSG00000115457 | IGFBP2 | 8.0 | 7.93338E-03 |
| ENSG00000239519 | CADM2-AS1 | 8.1 | 3.70903E-02 |
| ENSG00000264707 | L3MBTL4-AS1 | 8.1 | 7.93338E-03 |
| ENSG00000109193 | SULT1E1 | 8.1 | 2.63150E-02 |
| ENSG00000044524 | EPHA3 | 8.2 | 3.49563E-02 |
| ENSG00000164741 | DLC1 | 8.5 | 7.26942E-03 |
| ENSG00000263146 | LINC01896 | 8.5 | 8.39752E-03 |
| ENSG00000241186 | TDGF1 | 9.1 | 3.83781E-02 |
| ENSG00000251567 | AC018680.1 | 23.9 | 1.15310E-11 |
| ENSG00000153303 | FRMD1 | 23.7 | 1.15310E-11 |

**B.** Top 32 highest differentially expressed genes in the serum of OSCC vs. HC.

| **SERUM OSCC vs. HC** |  |  |  | |
| --- | --- | --- | --- | --- |
| **GeneID** | **GeneName** | **log2FC** | | **padj** |
| ENSG00000132376 | INPP5K | 27.8 | 4.3835E-18 | |
| ENSG00000163510 | CWC22 | 27.2 | 6.1414E-18 | |
| ENSG00000135919 | SERPINE2 | 27.4 | 1.9014E-17 | |
| ENSG00000111727 | HCFC2 | 27.1 | 3.2841E-17 | |
| ENSG00000161800 | RACGAP1 | 27.3 | 1.9014E-17 | |
| ENSG00000118520 | ARG1 | 27.3 | 1.9367E-17 | |
| ENSG00000061936 | SFSWAP | 27.5 | 1.5095E-17 | |
| ENSG00000145349 | CAMK2D | 27.0 | 6.486E-23 | |
| ENSG00000163687 | DNASE1L3 | 26.8 | 4.3032E-20 | |
| ENSG00000118260 | CREB1 | 26.5 | 7.4863E-20 | |
| ENSG00000173039 | RELA | 26.3 | 3.8672E-19 | |
| ENSG00000179152 | TCAIM | 26.8 | 8.0841E-18 | |
| ENSG00000197102 | DYNC1H1 | 26.9 | 9.2284E-18 | |
| ENSG00000089012 | SIRPG | 26.7 | 7.5281E-17 | |
| ENSG00000073756 | PTGS2 | 26.2 | 2.5924E-16 | |
| ENSG00000163739 | CXCL1 | 23.4 | 1.6688E-13 | |
| ENSG00000005381 | MPO | 22.2 | 2.9578E-12 | |
| ENSG00000087088 | BAX | 21.5 | 1.3345E-11 | |
| ENSG00000132470 | ITGB4 | -26.1 | 2.3186E-16 | |
| ENSG00000100100 | PIK3IP1 | -26.0 | 2.7195E-16 | |
| ENSG00000165410 | CFL2 | -26.7 | 8.0841E-18 | |
| ENSG00000166148 | AVPR1A | -26.5 | 1.5355E-20 | |
| ENSG00000198223 | CSF2RA | -27.3 | 1.8242E-17 | |
| ENSG00000047249 | ATP6V1H | -27.7 | 8.0841E-18 | |
| ENSG00000123575 | FAM199X | -28.0 | 4.6024E-18 | |
| ENSG00000151176 | PLBD2 | -27.9 | 4.6024E-18 | |
| ENSG00000072803 | FBXW11 | -27.6 | 9.7339E-18 | |
| ENSG00000136247 | ZDHHC4 | -27.6 | 2.6567E-19 | |
| ENSG00000187116 | LILRA5 | -27.7 | 9.9863E-23 | |
| ENSG00000137274 | BPHL | -28.3 | 2.7264E-18 | |
| ENSG00000184584 | TMEM173 | -28.1 | 3.7078E-18 | |
| ENSG00000214063 | TSPAN4 | -28.3 | 2.7264E-18 | |

**Supplementary Table S8.** Predicted targets of saliva and serum differentially expressed genes [Oral cancer (OC) vs. healthy individuals (HC)] through canonical pathways by IPA (HNC: head and neck cancer).

**A.** Saliva

| **SALIIVA mRNA signatures** |  |  |  |
| --- | --- | --- | --- |
| **Upstream Regulator (Function)** | **Molecule Type** | **Predicted Activation State (activation z-score, p-value)** | **Target Molecules in Dataset** |
| **IL33** (pre-tumorigenic) | Cytokine | Activated (2, 0.0307) | MMP10, PTPRK, SLC26A4,TP63 |
| **Alpha catenin** (tumor suppressor) | group | Inhibited (-2, 0.000363) | EPHA3, IGFBP2,TDGF1,TNC |
| **SALIIVA activated pathway** |  |  |  |
| **Tumor Microenvironment pathway** |  |  |  |
| **Upregulate genes in OC vs. HC** | **Location** | **Biomarker** | **Drug** |
| **TNC** (8, 0.036) | Extracellular |  | F16-IL2 fusion protein, Iodine I 131 monoclonal antibody F16SIP |
| **MMP-10** (6.8, 0.026) | Extracellular |  | Marimastat, certolizumab |
| **Tp63** (4.6, 0.027) | Nucleus | OC diagnosis |  |
| **Signaling related-molecules** | **Location** | **Biomarker** | **Drug** |
| VEGF | Extracellular | OC efficacy / HNC prognosis, disease progression, efficacy | bevacizumab/sorafenib, bavacizumab/cetuximab |
| MYC | Nucleus | OC efficacy |  |
| PTGS2 | Cytoplasm | OC efficacy | etoricoxib |
| MMP9 | Extracellular | HNC prognosis, efficacy | GS-5745 |
| MMP | Nucleus | HNC prognosis, efficacy | Certolizumab, GS-5745 |
| PDGF | Extracellular | HNC efficacy |  |
| NFkB | Nucleus | HNC efficacy | NF-kB inhibitor |
| BCL2 | Cytoplasm | HNC efficacy | afatinib/pacilitaxel, pacilitaxel/pembrolizumab/ramucimumab, AZD0466 |
| CCND1 | Nucleus | HNC efficacy |  |
| STAT3 | Nucleus |  | STAT3 inhibitor |
| IL1B | Extracellular | OC diagnosis |  |
| CXCL8 | Extracellular | OC diagnosis, HNC efficacy |  |
| IL6 | Extracellular | HNC prognosis, disease progression |  |
| RAS | Cytoplasm | HNC prognosis, response to therapy |  |
| CD44 | Plasma Member. | HNC prognosis |  |
| FGF | Extracellular | HNC efficacy |  |
| IGF | Extracellular | HNC efficacy |  |
| SLC2 | Plasma Membr. | HNC efficacy |  |
| TNF | Extracellular |  | infliximab/methotrexate, certolizumab |
| JAX2 | Cytoplasm |  | AZD-1480 |

**B.** Serum

| **SERUM mRNA signatures** |  |  |  |
| --- | --- | --- | --- |
| **Upstream Regulator (function / Expr Log Ratio)** | **Molecule Type** | **Predicted Activation State (activation z-score, p-value)** | **Target Molecules in Dataset** |
| CCAR2 (tumor promoting co-activator) | Peptidase | Activated (2.5, 0.000668) | CAMK2D, CREB1, DCAF4, MDM2, NR1D1, RAD50 |
| RELA (proto-oncogene) (26.3, p<0.00001) | Transcription regulator | Activated (2.9, 0.344) | CXCL1, DOCK8, GCH1, PTGER4, PTGS2, RELA, TLR2, TRAF1, ZFP91 |
| SIRT1 (tumor suppressor) | Transcription regulator | Inhibited (-2, 0.0339) | BAX, BNIP3, HSPA4, NAT1, NR1D1, PDCD6IP |
| **SERUM activated pathway** |  |  |  |
| **IL-8 signaling** |  |  |  |
| **Upregulated molecules in OC vs. HC** | **Location** | **Biomarker** | **Drug** |
| **KDR** (23, 5.34364783872724E-14) | Plasma Membrane | HNC efficacy |  |
| **CXCL1** (23, 1.66884E-13) | Extracellular |  |  |
| **MPO** (22, 2.95776717831948E-12 | Cytoplasm |  |  |
| **PTGS2** (26, 2.5924394621986E-16 | Cytoplasm | OC efficacy | etoricoxib |
| **BAX** (21, 1.33452501211123E-11 | Cytoplasm | HNC efficacy |  |
| **RELA** (26, 3.86723070798403E-19 | Nucleus |  | NF-kB decoy, dexamethasone/thalidomide, bortezomib/dexamethasone/thalidomide |
| **CXCL8** (23, 1.66884E-13) | Extracellular | OC diagnosis, HNC efficacy | lenvatinib |
| **Signaling related molecules** | **Location** | **Biomarker** | **Drug** |
| VEGFR | Plasma Membrane | HNC diagnosis, prognosis, efficacy, disease progression | anlotinib, lenvatinib, everolimus/vandetanib, bevacizumab/cetuximab, |
| EGFR | Plasma Membrane | HNC efficacy, response to therapy | everolimus/vandetanib, bevacizumab/cetuximab, afatinib/pacitaxel, afatinib/dasatinib |
| SRC | Cytoplasm | HNC prognosis, efficacy |  |
| BCL2 | Cytoplasm | HNC efficacy | afatinib/pacitaxel, afatinib/dasatinib, AZD0466 |
| BAX | Cytoplasm | HNC efficacy |  |
| MMP2 | Extracellular | HNC efficacy | rebimastat, marimastat |
| MMP9 | Extracellular | HNC diagnosis, efficacy | rebimastat, marimastat |
| VCAM1 | Plasma Membrane | HNC efficacy |  |
| BCL2-L1-A | Cytoplasm | OC efficacy |  |
| PTGS2 | Cytoplasm | OC efficacy | etoricoxib |
| BCL-XL | Cytoplasm | OC efficacy | AZD0466 |
| RAS | Cytoplasm | HNC prognosis, response to therapy |  |
| **NF-κB signaling** |  |  |  |
| **Upregulated molecules in OC vs HC** | **Location** | **Biomarker** | **Drug** |
| TLR2 (10.7, 0.005445814 | Plasma Membrane |  |  |
| KDR (-23, 5.34364783872724E-14 | Plasma Membrane |  |  |
| RELA (26, 3.86723070798403E-19 | Nucleus |  | NF-kB decoy, dexamethasone/thalidomide, bortezomib/dexamethasone/thalidomide |
| **Signaling related-molecules** | **Location** | **Biomarker** |  |
| IL1 | Extracellular space | OC diagnosis | **Drug** |
| CASP8 | Nucleus | OC diagnosis |  |
| MAPK8 | Cytoplasm |  | Cetuximab/encorafenib |
| TNF | Extracellular space |  | infliximab/methotrexate, dexamethasone/thalidomide, bortezomib/dexamethasone/thalidomide |
| GFR | Plasma Membrane | HNC prognosis, response to therapy, efficacy | dexamethasone/thalidomide, bortezomib/dexamethasone/thalidomide |
| RAS | Cytoplasm | HNC prognosis, response to therapy, efficacy | AZD4785 |
| PI3K | Cytoplasm |  | BAY1082439 |
| **PI3K/Akt pathway** |  |  |  |
| **Upregulated molecules in OC vs HC** | **Location** | **Biomarker** | **Drug** |
| PTGS2 (26, 2.5924394621986E-16 | Cytoplasm | OC efficacy | etoricoxib |
| MDM2 (9.6, 0.00012333319822441 | Nucleus |  | BI 907828, CGM097 |
| RELA (26, 3.86723070798403E-19 | Nucleus |  | NF-kB decoy, dexamethasone/thalidomide, bortezomib/dexamethasone/thalidomide |
| ITBG4 (-26, 2.31864922135523E-16) | Plasma Membrane |  | etaracizumab, vedolizumab |
| **Signaling related-molecules** | **Location** | **Biomarker** | **Drug** |
| Cytokine repector | Plasma Membrane | HNC prognosis | daclizumab |
| PTEN | Cytoplasm | HNC efficacy |  |
| RAS | Cytoplasm | HNC prognosis, response to therapy, efficacy | AZD4785 |
| BCL-XL | Cytoplasm | OC efficacy | AZD0466 |
| BCL2 | Cytoplasm | HNC efficacy | afatinib/pacitaxel, afatinib/dasatinib, AZD0466 |
| Tp53 | Nucleus | OC efficacy, HNC prognosis | BI 907828, CGM097 |
| CDKN1A | Nucleus | HNC diagnosis, efficacy |  |
| CDKN1B | Nucleus | HNC response to therapy, efficacy | E7386 |
| CCND1 | Nucleus | HNC efficacy |  |
| **Molecular mechanism in Cancer** |  |  |  |
| **Upregulated molecules in OC vs HC** | **Location** | **Biomarker** | **Drug** |
| RELA (26, 3.86723070798403E-19 | Nucleus |  | NF-kB decoy, dexamethasone/thalidomide, bortezomib/dexamethasone/thalidomide |
| CAMK2D (29, 6.48599316123655E-23 | Cytoplasm |  | K-252, lavendustin C |
| MDM2 (9.6, 0.00012333319822441 | Nucleus |  | BI 907828, CGM097 |
| BRCA1 (-24, 2.89268361193085E-14) | Nucleus |  |  |
| ITBG4 (-26, 2.31864922135523E-16) | Plasma Membrane |  | etaracizumab, vedolizumab |
| **Signaling related-molecules** | **Location** | **Biomarker** | **Drug** |
| P53 | Nucleus | OC efficacy, HNC prognosis | BI 907828, CGM097 |
| CDKN2A | Nucleus | OC efficacy |  |
| BCL2-L1 | Cytoplasm | OC efficacy |  |
| RAS | Cytoplasm | HNC prognosis, response to therapy, efficacy | AZD4785 |
| SRC | Cytoplasm | HNC prognosis, efficacy |  |
| RB1 | Nucleus | HNC prognosis |  |
| CDH1 | Plasma Membrane | HNC prognosis, efficacy |  |
| CDKN1B | Nucleus | HNC response to therapy, efficacy | E7386 |
| NF-kB | Nucleus | HNC efficacy | NF-kB inhibitor, NF-kB decoy, dexamethasone/thalidomide, bortezomib/dexamethasone/thalidomide |
| CCND1 | Nucleus | HNC efficacy |  |
| CDKN1A | Nucleus | HNC diagnosis, efficacy |  |
| CDKN2A | Nucleus | HNC diagnosis |  |
| Cyclin D, E | Nucleus | HNC efficacy |  |
| NBN | Nucleus | HNC efficacy |  |
| HIF1alpha, HIF1A-NICD | Nucleus | HNC efficacy | BAY 87-2243 |
| CASP3/6/7 | Cytoplasm | OC diagnosis, HNC efficacy | Caspase 3 inhibitor |
| MYC, Max-Myc | Nucleus | OC diagnosis | AVI-4126 |
| HIF1alpha, HIF1A-NICD | Nucleus | HNC efficacy | BAY 87-2243 |
| **Tumor Microenviroment pathway** |  |  |  |
| **Upregulated molecules in OC vs HC** | **Location** | **Biomarker** | **Drug** |
| PTGS2 (26, 2.5924394621986E-16 | Cytoplasm | OC efficacy | etoricoxib |
| RELA (26, 3.86723070798403E-19 | Nucleus |  | NF-kB decoy, dexamethasone/thalidomide, bortezomib/dexamethasone/thalidomide |
| **p53 signaling** |  |  |  |
| **Upregulated molecules in OC vs HC** | **Location** |  |  |
| BAX (21, 1.33452501211123E-11 | Cytoplasm | HNC efficacy |  |
| MDM2 (9.6, 0.00012333319822441 | Nucleus |  | BI 907828, CGM097 |

**Supplementary Table S9. A.** *RELA(p65)* mRNA levels by mRNA-seq (normalized counts, by DESeq2) and RT-qPCR (normalized levels by *h*GAPDH) in the serum of OSCC patients. **B.** Significantly differentially expressed genes in the saliva of OSCC patients with a smoking history versus healthy smokers.

**A.**

| ***RELA(p65)* expression in SERUM OSCC** | | |
| --- | --- | --- |
| **mRNA-seq** | RT-qPCR |  |
| 2931.6 | 3.37000 |  |
| 877.7 | 0.21997 |  |
| 771.6 | 0.01824 |  |
| 26.2 | 0.02181 |  |
| 4.8 | 0.00310 |  |
| 2.5 | 0.03949 |  |
| 2.5 | 0.01152 |  |

**B.**

| **SALIVA OSCC SM vs HC SM** | | | |
| --- | --- | --- | --- |
| **GeneID** | **GeneName** | **log2FC** | **padj** |
| ENSG00000279319 | AC105074.1 | 23.7 | 2.9362E-10 |
| ENSG00000183801 | OLFML1 | 24.2 | 2.0033E-10 |
| ENSG00000185518 | SV2B | 23.9 | 2.6077E-10 |
| ENSG00000153303 | FRMD1 | 23.4 | 3.5748E-10 |
| ENSG00000263503 | MAPK8IP1P2 | -26.1 | 9.7277E-13 |

**Supplementary Fig. S1.** Saliva differentially expressed genes in OSCC vs. HC are associated with tumor micro-environment pathways.****

**Supplementary Fig. S2.** Serum differentially expressed genes in OSCC vs. HC are associated with IL-8 Signaling Pathway.

**Supplementary Fig. S3.** The diagram represents the predicted interactions between saliva miR-187-3p and saliva mRNAs, associated with tobacco smoke-related OSCC, through the mTOR canonical pathway and its predicted targets, by IPA. The table presents saliva miR-187-3p, its location, predicted targets, function, pathway, and drugs that can also affect its target.
